# Supplementary material for: Nanostructured polymer films with metal-like thermal conductivity
Source: Nat Commun. 2019 Apr 16;10:1771. doi: 10.1038/s41467-019-09697-7 (PMC6467866; doi:10.1038/s41467-019-09697-7)
Supplement: Supplementary file 1 — Supplementary Information [file 41467_2019_9697_MOESM1_ESM.pdf]

Supplementary Information for

Nanostructured Polymer Films with Metal-like Thermal Conductivity

Yanfei Xu et al.

Supplementary Note 1. Fabrication of thermally conductive polyethylene films

Polyethylene solution was prepared by adding ultrahigh molecular weight polyethylene powders (UHMWPE, molecular weight 3-6 million Da, 3 wt%) into decalin solvent, and was heated to 145 °C for 24 hours in a silicone oil bath. Subsequently, the hot solution was extruded onto a liquid nitrogen-cooled glass substrate using a high-shear-rate Couette-flow extrusion system[1]. The majority of solvent was evaporated at ambient conditions for ~36 hours. The as-extruded films were then removed from the substrate and fed to a constant-force adaptive thickness roll-to-roll drawing platform (heated, ~90 °C)[1]. The draw ratios were obtained as the ratio of final to initial film length, with ~20% uncertainty.

Supplementary Note 2. Steady-state thermal conductivity measurements

Thermal conductivity measurement of the plastic films is very challenging because the samples are very thin at high draw ratio (~1-3 microns). A recent experimental study[2] of Dyneema, Zylon and Spectra fibers suggested that some previous work may have overestimated thermal conductivities by as much as a factor of 3. We explored numerous methods to measure thermal conductivity of the drawn films, and eventually decided to use an extensively validated home-built steady-state platform[3–5]. We calibrated the setup by measuring various control samples, including 304-stainless steel foils[6], Dyneema fibres[2] and Zylon fibres[2], Sn[7] and Al[7] films, and measured thermal conductivity 15.3 W m^{-1} K^{-1}, 22.6 W m^{-1} K^{-1}and 23.6 W m^{-1} K^{-1}, 64.4 W m^{-1} K^{-1} and 202.7 W m^{-1} K^{-1} for 304-stainless steel foils[6], Dyneema fibres[2] and Zylon fibres[2] , Sn[7] and Al[7] films, respectively. These measured thermal conductivity is similar to those reported in previous references[2,6,7]. We also used a transient pump-probe technique[8–10] to measure a thick laminate and the results are consistent with the steady-state method[3–5]. Each method is described in more detail below.

Thermal conductivity measurement principle of steady-state method: Direct measurement of the electrical heating power (P_{el}) as a function of temperature difference ($T_h - T_c$) across a sample film was performed[3] (Supplementary Figure 1). T_h (303 K) was kept constant via feedback control of P_{el}, while T_c was reduced to create a small temperature difference (up to 10 K) by systematically increasing the thermoelectric cooling power. Multiple measurements of P_{el} were performed at a given temperature difference once the system had reached steady state (Supplementary Figure 1b). Subsequently, the slope of the linear fit yields, according to Fourier's law and after correction for thermal shunting (Supplementary Figure 1b). Convection and parasitic heat loss were minimized using high vacuum and a temperature-controlled copper shield,

respectively. Special effort was taken to minimize the thermal radiation exchange and to ensure that the reported thermal conductivity is conservative even if any residual radiation exists. Thermal shunting was minimized and quantified after each experiment by removing the film sample and repeating the measurement.

We create a geometry such that the one-dimensional (1D) Fourier law of heat conduction is satisfied[3–5], which is shown in Supplementary Equation 1.

$$Q = \frac{kA}{L}(T_h - T_c) = \frac{kA}{L}\Delta T \tag{S.1}$$

where $A,\ k,\ L,\ T_h$ and T_c, Q are the sample cross-sectional area, thermal conductivity, sample length, hot side temperature, cold side temperature and heat flow through the sample, respectively. Figure 2a and Supplementary Figure 1a show a schematic and some images of the experimental setup, respectively. The sample was suspended between a hot junction clamp and a cold junction clamp, all of which were guarded by a copper radiation shield. We maintained the hot clamp temperature T_h constant using a resistive electrical heater while the cold clamp temperature T_c was systematically lowered by a thermoelectric cooler. We achieved this by feed-back controlling the electrical heating power P_{el} into the heater as the cooling power increased (Figure 2a, Figure 2b and Supplementary Figure 1).

Analysis of the parasitic heat losses: By keeping the hot clamp and copper radiation shield at the same constant temperature, parasitic heat losses such as through the electrical leads to the heater and thermocouples were effectively kept constant, which therefore did not affect the slope of P_{el} versus the temperature differential ΔT. As shown in Fig. 2b and Supplementary Figure 1b, Supplementary Figure 1c and Supplementary Figure 1i, all our sample measurements and

calibration measurements showed a linear behavior. The thermal conductivity of the sample was obtained from Supplementary Equation 2.

$$k = \frac{L}{A} \times \frac{dP_{el}}{d(\Delta T)} \tag{S.2}$$

Analysis of the uncertainties due to thermal radiation: This method only leads to accurate results when the surrounding temperature is constant to ensure constant parasitic heat losses from the hot side and radiation loss is minimal. For these purposes, a temperature-controlled copper radiation shield guarded the heater clamp and the suspended sample. The radiation shield was constantly maintained at the hot side temperature, T_h, and all heater current leads and hot side thermocouple wires were thermally grounded to minimize parasitic heat losses which was especially important for samples with small thermal conductance.

Parasitic radiation heat transfer between the sample and the environment is of major concern for the accuracy of the thermal conductivity measurements. Two measures were taken to ensure the reliability of reported data. As already mentioned, the sample was surrounded by a temperature-controlled (at T_h) radiation shield. This configuration led to an additional heat input from the shield into the suspended sample, which reduced the required electrical heating power P_{el} for a given temperature difference and hence the reported thermal conductivity is a more CONSERVATIVE value. Second, the sample length was chosen to ensure that heat conduction along the sample is higher than the surface radiation loss (Supplementary Figure 2d). The ratio of radiation to conduction heat flow can be approximated as:

$$\frac{Radiation}{Conduction} = \frac{L^2 \varepsilon \sigma ((3T_h + T_c)/2)^3}{kt} \tag{S.3}$$

where t is the sample thickness, k the thermal conductivity, L the sample length, ε the emittance, σ the Stefan-Boltzmann constant, T_h and T_c the hot and cold side temperatures of the sample.

We attempted to measure emittance based on reflectance and transmittance values, but the low emittance and experimental uncertainties sometimes led to negative values. Therefore, we decided to use emittance of the films at different draw ratios (different thickness) based on the following empirical formula found in literature[11]:

$$\varepsilon = 2.51 \times 10^{-3}t - 3.12 \times 10^{-6}t^2 + 4.55 \times 10^{-6}tT - 4.13 \times 10^{-4}T + 0.206 \qquad \text{(S.4)}$$

where T is the film temperature in Celsius, and t is the sample thickness in microns which were carefully measured using a micrometer and a profilometer, as detailed in the section below (Supplementary Figure 2a and Supplementary Figure 2b).

Supplementary Figure 2c shows the films' emittance at 298 K based on Supplementary Equation 4. Supplementary Figure 2d shows the right-hand side of Supplementary Equation 3 for the measured films at different draw ratios. In general, radiation errors are less than 20%, and for the higher draw ratios, less than 10%.

We analyze the radiative thermal shunting loss here. We carefully considered thermal radiative shunting between the hot and cold side. We minimized radiative shunting by using polished copper ($\varepsilon < 0.05$) for the hot and cold clamps (Supplementary Figure 1a). In addition, radiative thermal shunting calibration was performed (Supplementary Figure 1f). The same measurement procedure was used without a suspended sample allowing to obtain the radiation heat flow between the hot and the cold side. To ensure a large enough signal-to-noise ratio, the sample geometries were optimized such that the radiative thermal shunting is limited to less than

20% relative to heat conduction by the sample, and less than 10% for most samples (Supplementary Figure 1d, Supplementary Figure 1e, and Supplementary Figure 1f).

Analysis of uncertainties due to sample geometry: error in determining the drawn film thickness was minimized by using a Bruker DektakXT stylus profilometer, which was first calibrated by a 45-nm step height standard from Bruker company. The drawn film was mounted on a silicon wafer, and the film thickness was obtained by the edge step. Supplementary Figure 2a shows film thicknesses at different draw ratios. Each film thickness was measured 10 times at different locations along the sample. Supplementary Figure 2b shows some representative thickness profiles for the 110× film. The thicknesses of the drawn polyethylene films from 10× to 110× are ~$1-8\ \mu m$, which were measured using a profilometer. The as-extruded (1×), 2.5× and 5× film thicknesses are 76.4 μm, 16.5 μm, and 14.3 μm, respectively, as measured using a micrometer. Length and width of the film were measured after it was suspended between the clamps. Briefly, we first took a picture of the film and clamps under a microscope. The precisely machined heater clamp provides a scale bar to which the sample length is compared. This gives a reasonable accuracy of $\pm 50\ \mu m$. Measured sample dimensions including the lengths and cross-sectional areas are listed in Supplementary Table 1 and Supplementary Table 2.

Platform calibration: prior to measuring the polyethylene films, we measured control samples including 304-stainless steel foils[6], Zylon fibres[2], Dyneema fibres[2], Sn[7] and Al[7] films, obtaining thermal conductivities of ~15.3 (+1.1, −0.6) W m^{-1} K^{-1}, ~22.6 (+5.0, −4.5) W m^{-1} K^{-1}, ~23.6 (+4.4, −3) W m^{-1} K^{-1}, ~64.4 (+5.7, −5.4) W m^{-1} K^{-1}, ~202.7 (+15.6, −15.1) W m^{-1} K^{-1}, respectively, which are in good agreement with literature values[2,6,7]. Here, the thickness of the 304-stainless steel foils was measured by a micrometer to be 50 μm, and the cross-sectional area was 0.47 mm^2. The Zylon sample is a bundle of 664 individual fibers. The Dyneema sample

consists of 300 fibers. The average diameter of a single Dyneema fiber and Zylon fiber are 17 μm and 11.7 μm, respectively, which were characterized with SEM (Supplementary Figure 3). The estimated radiation to conduction ratio for the measured Dyneema bundle was <2%.

Minimization of the thermal interface resistance: temperatures of the hot and cold sides were measured by thermocouples attached to the hot and cold clamps. Key to accurately measuring the temperatures is the use of proper thermal interface materials between the film and the copper clamps. Several thermal interface materials (TIM) were investigated using the 304-stainless steel foils[6], Dyneema fibres[2] and Zylon fibres[2] as the reference samples, which has similar thermal conductance as our polymer films. We found that a certain type of unhardened silver epoxy paste provided a reliable contact and the smallest interface thermal resistance, as compared to other TIMs such as indium foil or silicone-based thermal paste. Using the unhardened silver epoxy paste also gave us the opportunity to disassemble the setup without damaging the sample and with some epoxy left on the clamped ends of the sample as marks. The distance between the marks corresponds to the length of the sample and allows us to double-check our initial length measurement (via the microscope image). We minimize thermal interface resistance by using thermal paste as much as possible (Supplementary Figure 1a). Because there is an effect of thermal contact resistance, our measurement tends to UNDERESTIMATED thermal conductivity of all the samples including drawn polyethylene films.

Effect of the anisotropic thermal conductivity of the films: one of the main characteristic properties of the drawn polymer films is the large anisotropy in thermal conductivity. In the in-plane draw direction the thermal conductivity (k_{IP}) is drastically larger than the perpendicular directions such as the cross-plane thermal conductivity (k_{CP}). This inevitably affects the temperature profile in the clamped region as shown schematically in Supplementary Figure 4a.

Mean sample temperature to the left of the cold clamp can be significantly higher compared to the clamp temperature. In order to estimate the effect we approximate the two-dimensional (2D) fin conduction problem with a 1D fin problem by lumping the sample's cross-plane thermal resistance into an effective thermal contact resistance $r_{th,c} \approx t/4k_{CP}$ (Supplementary Figure 4b). The resulting differential fin equation is

$$k_{IP}\frac{t}{2}\frac{d^2T}{dx^2} - \frac{k_{CP}}{t/4}(T - T_c) = 0 \tag{S.5}$$

with t being the film thickness, T and T_c being the sample temperature along x-direction and the cold clamp temperature, respectively. The differential equation can be solved with a specified heat flux, q, at the left side of the clamp and the sample temperature being T_c at infinity as the two boundary conditions.

$$T(x) = T_c + \frac{qt}{\sqrt{2k_{IP}k_{CP}}}e^{-\sqrt{\frac{k_{CP}}{k_{IP}}\cdot\frac{8}{t^2}}x} \tag{S.6}$$

The introduced error in the thermal conductivity measurement stems from the difference in the copper clamp temperatures compared to the actual sample temperature at the beginning of the clamp. The simulation results show that a significant error is only introduced for thick films with large thermal conductivity anisotropy (Supplementary Figure 4c). The measurements of single layer films with thicknesses ~1− 8 μm (Supplementary Figure 2a) are not significantly affected even for thermal conductivity anisotropy ratios above 200.

Supplementary Note 3. Time-domain thermoreflectance measurements

Sample preparation: One of the most challenging steps in the time-domain thermoreflectance (TDTR) experiments is sample preparation, whereby a flat and smooth cross-sectional surface of the drawn film has to be created[12]. To this end, we hot pressed (Carver 4120) 100 layers of 50× films into a laminate (~150 µm thick, 1 mm wide and 2 cm long) at 120 °C for 40 minutes. We did not expect dramatic structural change due to the elevated temperature, since the film melting point was measured using differential scanning calorimetry (TA Instruments Discovery) to be ~140 °C (Supplementary Figure 5a), consistent with previously reported values (~144 °C) for UHMWPE[13]. We further embedded the laminated 50× film into an epoxy matrix, which was necessary for us to properly mount and cut the cross-section using a microtome (Leica Microsystems). This cutting procedure created a flat and smooth cross-section surface of the laminate and the surrounding epoxy. Using atomic force microscope characterization, the root-man-square roughness of the cross-section surface was measured to be ~10 nm in a 15 µm × 15 µm region (Supplementary Figure 6). It is quite challenging to obtain such ultra-smooth (root-mean-square roughness ~ 10 nm) and aligned polymeric films without altering the sample. The difficulty in sample preparation for TDTR is further complicated by the fact that the yield of our drawing platform is not very uniform at higher draw ratio. We thus only prepared one thick sample for the TDTR experiment.

Measured thermoreflectance signals were fitted to a standard two-dimensional, 3-layer heat conduction model considering the aluminum (Al) transducer, the laminate and in-between interface. Both the out-of-plane (film draw direction) and in-plane thermal conductivity of the UHMWPE laminate were explicitly modeled to account for the expected anisotropy.

Laser parameters: a 100-fs-wide pump laser pulse (~400 nm center wavelength) was used to instantly heat up the surface of an aluminum-coated sample (Supplementary Figure 6), the cooling of which was then monitored using a probe pulse (800 nm) as a function of delay time between the pulses (Fig. 2c)[9,10]. Subsequently, the cooling curves were fitted to a standard two-dimensional heat transfer model to get the sample thermal conductivity (Fig. 2c and Fig. 2d). In order to increase the signal-to-noise ratio, modulated heating was applied by electro-optical modulation of pump power, which resulted in a complex signal with its amplitude and phase recorded by a lock-in amplifier. Both the amplitude and phase signals were used for model fitting. The excellent agreement between amplitude and phase fitting confirmed the measurement reliability (Fig. 2d and Supplementary Figure 7). Changing the fitted thermal conductivity by 20% led to a large discrepancy between the simulated and measured curves, further indicating good experimental sensitivity (Fig. 2d and Supplementary Figure 8). The reported value in Fig. 3 was obtained as an average of 20 experiments at 3 MHz and 6 MHz modulation.

The incident pump beam power was varied from about 20 mW to 50 mW across multiple measurements, with no pump-power dependence observed. The incident probe power was kept at ~6 mW. At room temperature, such power levels typically result in a small (~1 K) temperature increase so that the change in aluminum reflectance remains proportional to its temperature variation. The $1/_{e^2}$ diameters of the pump and probe beams were measured to be 53 μm and 11 μm, respectively, using a scanning slit beam profiler. Such a configuration ensures that the thermoreflectance signal essentially captures a 1D heat conduction process perpendicular to the sample surface, or equivalently, parallel to the draw direction (Fig. 2c). This setup also helps minimize any uncertainty associated with inaccurate measurement of laser beam size. Finally,

multiple pump modulation frequencies (3 MHz and 6 MHz) were used to see if there is any frequency dependence.

Aluminum transducer layer thickness: the Al layer thickness was set as 90 nm during electron-beam evaporation and was subsequently measured using a profilometer as 88 ± 3 nm. Thickness was further verified by performing TDTR measurement of a standard sapphire sample, which was Al-coated together with the UHMWPE sample.

Heat capacity of 50× films: differential scanning calorimetry measurement of the heat capacity of the 50× films was performed using a TA Instruments Discovery. The calorimeter was calibrated with a standard sapphire sample prior to measurement of the films. The sample temperature was cycled 3 times between 180 K and 340 K at a heating rate of 5 K/min. The measured specific heat (Supplementary Figure 5b) agrees well with literature values[14], especially near room temperature. Further, a density of 0.97 g cm^{-3} was used for conversion to volumetric specific heat[15].

Measured thermal conductivity and interface conductance: thermal conductivity of the laminate (100 layers of 50× films) along the draw direction was measured as 33.6 ± 4.5 W m^{-1} K^{-1}, in excellent agreement with values obtained using the steady-state method (Fig. 3b). The Al/UHMWPE interface thermal conductance was simultaneously fitted to be 44.7 ± 4.0 MW m^{-2} K^{-1}, which is typical for smooth interfaces at room temperature. In comparison, thermal conductance of a smooth Al/sapphire interface is usually 90 MW m^{-2} K^{-1}. The above-mentioned values are averages of 10 individual runs at 3 MHz pump modulation and another 10 at 6 MHz (Supplementary Figure 7). When averaged separately, the 3 MHz data yield a film conductivity of 34.8 ± 5.3 W m^{-1} K^{-1} and an interface conductance of 45.3 ± 5.1 MW m^{-2} K^{-1}, while the 6 MHz

data yield 32.3 ± 3.1 W m^{-1} K^{-1} and 44.1 ± 2.5 MW m^{-2} K^{-1}, essentially indicating no frequency dependence. We have also verified through sensitivity analysis that both amplitude and phase fitting are reliable for the current study (Supplementary Figure 8). Further, the anisotropy ratio of in-plane to out-of-plane thermal conductivity was investigated to have a negligible effect (<1%) on the fitting results.

Supplementary Note 4. Structural characterization with synchrotron X-ray

Synchrotron X-ray scattering measurements were carried out at Sector 8-ID-E of Advanced Photon Source (APS), Argonne National Laboratory, with an unfocused and collimated 10.91 keV X-ray beam of cross-section 200×200 μm^2 (V×H). Samples were measured in a vacuum chamber in order to minimize radiation damage and scattering background from air and X-ray windows. Single-photon-counting area detector Pilatus 1MF was mounted 137 mm and 2153 mm downstream away from the sample for wide-angle X-ray scattering (WAXS) and small-angle X-ray scattering (SAXS), respectively. Each sample was translated for multiple examination to ensure macroscopic structural homogeneity. Raw WAXS and SAXS patterns were processed with various corrections with MATLAB-based GIXSGUI software before quantitative structural analysis. The film surface was perpendicular to the incident beam as shown Fig. 4a.

WAXS and SAXS data corrections: intensity of the 2D WAXS and SAXS images was corrected on a pixel-by-pixel basis[16]:

$$I_{corrected} = I_{raw} \frac{E_m E_d F C_S}{PL} \tag{S.7}$$

where I_{raw} is the raw data, E_m is the air gap absorption correction, E_d is the detector efficiency correction, F is the detector's flat-field correction at the operation energy of 10.91 keV, C_S is the solid angle correction, P is the polarization correction, L is the Lorentz correction.

WAXS analysis: We identified an orthorhombic cell with lattice constants a = 7.42 Å, b = 4.95 Å and c = 2.54 Å, which agreed well with reference values[17].

Effective crystallinity: X-ray diffraction (XRD), or WAXS, is routinely employed to measure the percentage of crystallinity of materials consisting of crystalline and amorphous

components[18]. It is based on the assumption that the number of elastically scattered photons by one phase is proportional to the amount of that phase in the scattering volume. This leads to another requirement for this method to work, i.e. tagging each scattered photon to the corresponding phase (crystalline or amorphous). The tagging of photons can be readily achieved provided a known crystalline lattice type and structure[18]. As given in the Supplementary Equation 8, the crystallinity is calculated as the ratio of the integrated intensity from the crystalline peaks to the sum of the crystalline and amorphous intensities[18].

$$Crystallinity = \frac{I_{crystalline}}{I_{crystalline} + I_{amorphous}} \tag{S.8}$$

Most samples for crystallinity analysis often take the form of powders. It is quite a challenge to obtain the true value of crystallinity from polymer fiber or sheet samples using this method[19]. This is because while the amorphous components are isotropic, ordered polymer chains and crystalline components often adopt certain orientations, nullifying the isotropy assumption of the method.

In this work, we introduce a concept of "effective crystallinity" which attempts to approximate the true crystallinity. It was calculated with the same Supplementary Equation 8 but on a single WAXS image. Since identical WAXS geometry was employed with an area detector for all samples in this work, the trend of the change of the degree of the crystallinity can be qualitatively analyzed as the draw-ratio increases. The limited active area of the detector captures only a little over a quarter of the entire WAXS pattern, i.e. the {*hk0*} and {*hk1*} Bragg groups (Supplementary Figure 9a and Supplementary Figure 12). Considering the symmetry of the scattering which contains four {*hk1*} groups and two {*hk1*} groups, when converting to 1D

scattering profile, we are able to extend to the entire WAXS pattern with four quarters by double counting the region containing the $\{hk1\}$ group (Supplementary Figure 9a).

The 1D intensity profile was then fitted to the sum of a series of Voigt functions (which is a convolution of Gaussian and Lorentzian functions) to represent each amorphous and crystalline peak, plus a linear background (Supplementary Figure 9b, 5× as an example). The integrated intensities of the crystalline peaks were then individually calculated and summed before division by the total intensity to get effective crystallinity. Following such a procedure, an average effective crystallinity of 0.67 was obtained for the 2.5× films, and a crystallinity as high as 0.92 for the 110× films (Fig. 4d and Supplementary Figure 9f). The amorphous fraction of 0.33 was obtained for the 2.5× films, and the amorphous fraction of 0.08 for 110× films (Fig 4f). We note that on high draw-ratio samples, for example, as in the 110× sample, while unprocessed 2D pattern does not reveal significant amorphous contribution, sufficient photon statistics can be seen after the data is converted to 1D XRD curve and thus a high confident estimation of the crystallinity is warranted. We note that considerable improvements have been achieved over the years both in the film quality and in our X-ray scattering measurements and analysis[1].

Crystallite orientation: the degree of orientation was quantified by orientation order parameters defined as the intensity-weighted moments of $\cos\beta$, where β is the tilt angle between the c-axis and draw direction (Fig. 4a)[20]. The orientation orders can be calculated directly from the measured azimuthal intensity distributions. Perfect alignment of the crystallites results in $\langle \cos^n \beta \rangle = 1$, where n is the order of the moment (Fig. 4d and Supplementary Figure 9c).

During stretching, the c-axis of the crystallites (Fig. 4a, chain direction) tends to align with the draw direction. Since the a, b, c axes (Fig. 4a) are mutually orthogonal for standard

orthorhombic unit cells, and there is no evidence that the *a* and *b* axes prefer any orientations except being orthogonal to the *c*-axis, we thus can determine the orientation order from the azimuthal intensity profiles $I(\phi)$ across the $\{hk0\}$ peaks via an analytical, closed-form method that was developed for liquid crystals[20]. In this method, the 1st, 2nd and 4th order parameters (Supplementary Figure 9c) are given by

$$P1 = N^{-1}\int_0^{\pi/2} I(\o)(cos^2\o)d\o \tag{S.9}$$

$$\bar{P}2 = 1 - N^{-1}\frac{3}{2}\int_0^{\frac{\pi}{2}} I(\o)\{sin^2\o + (sin\o)(cos^2\o)ln[(1+sin\o)/cos\o]\}\,d\o \tag{S.10}$$

$$\bar{P}4 = 1 - N^{-1}\frac{3}{2}\int_0^{\frac{\pi}{2}} I(\o)\left\{sin^2\o(\frac{105}{16}cos^2\o + \frac{15}{24}) + (sin\o)ln[(1+sin\o)/cos\o](\frac{105}{16}cos^4\o - \frac{15}{4}cos^2\o)\right\}d\o \tag{S.11}$$

where $N = \int_0^{\frac{\pi}{2}} I(\o)d\o$ is the normalization constant (Supplementary Figure 9d). Since all $\{hk0\}$ peaks resulted in identical values for the order parameter as we expected, these values were then averaged to give the overall order parameters.

SAXS analysis: SAXS technique is capable of discovering the structural information of a sample which has spatial inhomogeneities ranging from a few to several hundreds of nanometers. This method is often measured against the scattering wave vector transfer **q**, and the scattering intensity is in principle the modulus square of the Fourier transform of the spatial distribution of the electron density. In kinematic approximation[21], this relation is $I(\vec{q}) \sim \left|\int d\vec{r}\rho(\vec{r})e^{-i\vec{q}\cdot\vec{r}}\right|^2$. The contrast of the electron densities between the crystalline and amorphous regions of the polyethylene films, although weak, is sufficient to create enough SAXS signals for the structure analysis. The humps in the SAXS intensity profile along the draw direction signal a periodic structure along that direction; and the **q** positions of the humps scale as 1:2 (Fig. 4e), suggesting a

layered or lamellar superlattice packing[21] (alternating crystalline and amorphous phases) with its repeating unit schematically depicted as Supplementary Figure 10 and Supplementary Figure 11a.

The term "amorphous" often refers to those non-crystalline and much disordered regions in a bulk material, where molecules have no preferred orientations. Upon mechanical stretching, molecules in an amorphous region may adopt certain orientations but remain in a non-crystalline state. Although it is changeling to distinguish this so-called "oriented amorphous" from totally randomly oriented amorphous regions directly from X-ray scattering data, their existence is able to be indirectly verified by the significantly improved thermal conductivities based on the thermal modeling (Supplementary Note 5) using the information of the statistical size analysis of X-ray scattering data from the crystalline region and its neighboring regions (random amorphous and/or oriented amorphous).

Lamellar superlattice structure: each repeating unit is modeled as a crystalline layer, an amorphous layer, and two transition layers (Supplementary Figure 10a). Since electron density in the transition layers is expected to vary from that of a crystallite to that of the amorphous, it is then reasonable to simplify the model to a two-phase unit of lengths L' and $L - L'$ (Supplementary Figure 10a). Rather than assuming a sharp interface as in a three-phase model, the transition between these two phases is effectively modeled as an interfacial roughness taking the form of an error function whose derivative is a Gaussian with a standard deviation denoted by σ. This model creates a smooth transition between crystal and amorphous layers, which is more realistic than the two-step functions in the three-phase model (Supplementary Figure 10).

Electron density profile: the electron density profile in each repeating unit along the c-axis (Supplementary Figure 10b) is thus a smooth profile $\rho(x)$ determined by L, L' and σ as

$\rho(x) = \frac{\sum_{j=1}^{3} \rho_j W_j(x)}{\sum_{j=1}^{3} W_j(x)}$, where $\rho_1 = \rho_3 = \rho_A$ and $\rho_2 = \rho_C$ are the electron densities of the amorphous and crystalline phases, respectively, and

$$W_1(x) = \frac{1}{2}\left[1 + \mathrm{erf}\left(\frac{x - \frac{L'}{2}}{\sqrt{2}\sigma}\right)\right] \tag{S.12}$$

$$W_2(x) = \begin{cases} \frac{1}{2}\left[1 + \mathrm{erf}\left(\frac{x}{\sqrt{2}\sigma}\right)\right], & x \le 0 \\ \frac{1}{2}\left[1 - \mathrm{erf}\left(\frac{x}{\sqrt{2}\sigma}\right)\right], & x > 0 \end{cases} \tag{S.13}$$

$$W_3(x) = \frac{1}{2}\left[1 - \mathrm{erf}\left(\frac{x + \frac{L'}{2}}{\sqrt{2}\sigma}\right)\right] \tag{S.14}$$

The Fourier transform of $\rho_L(x)$ is called the form factor in SAXS

$$F(q) = \int dx \rho(x) e^{-iqx} \tag{S.15}$$

A Gaussian distribution with a standard deviation of $\sigma_{L'}$ for the length L' is introduced to account for its polydispersity (Supplementary Figure 10b),

$$g(L'_\alpha) = \frac{1}{\sqrt{2\pi\sigma_{L'}^2}} \exp\left[-\frac{(L'_\alpha - L')^2}{2\sigma_{L'}^2}\right] \tag{S.16}$$

Structure factor and size distribution: the structure factor $S(q)$ describing how the units are stacked to form a lamellar superlattice, is modeled within the framework of the 1D para-crystal model[22], so that the long-range ordering is gradually destroyed in a probabilistic manner (which is often modeled as a Gaussian)[23]. This model allows a link between perfectly ordered and disordered structures, and provides a good analytical description of a structure in a partially ordered state. In

this 1D para-crystal model[24], the distance between successive units is independent of other distances and obeys a statistical distribution function $p(x)$ with $\int_{-\infty}^{\infty} dxp(x) = 1$. Assuming a Gaussian probability density, we have

$$p(x) = \frac{1}{\sqrt{2\pi\sigma_D^2}} \exp\left[-\frac{(x-D)^2}{2\sigma_D^2}\right] \tag{S.17}$$

whose Fourier transform is

$$P(q) = \exp(-q^2\sigma_D^2/2)\exp(iqD) \tag{S.18}$$

The autocorrelation function $g(x)$ of the positions of the superlattice units is given by

$$g(x) = \delta(x) + g^+(x) + g^-(x) \tag{S.19}$$

where $\delta(x)$ is the Dirac delta function, $g^+(x)$ and $g^-(x)$ are the autocorrelation functions on the positive ($x > 0$) and negative ($x < 0$) sides of the axis, respectively, which are written as

$$g^+(x) = p(x) + p(x) \otimes p(x) + p(x) \otimes p(x) \otimes p(x) + \cdots for\ x > 0 \tag{S.20}$$

and by symmetry

$$g^-(x) = g^+(-x)\ for\ x < 0 \tag{S.21}$$

Here $\otimes$ denotes the convolution. The structure factor is defined as the Fourier transform of $g(x)$ and thus given by

$$S(q) = 1 + P(q) + P^2(q) + P^3(q) + \cdots = \text{ Real}\left[\frac{1+P(q)}{1-P(q)}\right] \tag{S.22}$$

For lamellar superlattice stacking, $D = L$, and the distance of successive units along the drawing direction is coupled with the unit lengths, rather than being independent as in the pure 1D para-crystal model. We therefore adopt the size-spacing coupling approximation (SSCA)[24,25], which includes a size-spacing coupling parameter κ with $\kappa = 0$ automatically reduces to the decoupling approximation given in the classical para-crystal model. The structure factor in SSCA is

$$S(q) = 1 + 2\text{Real}\left\{\frac{\tilde{P}_\kappa^2(q)\Omega_\kappa(q)}{\tilde{P}_{2\kappa}(q)[1 - \Omega_\kappa(q)]}\right\} \tag{S.23}$$

with the scattering intensity at $q \neq 0$ given by

$$I(q) \sim |\langle F(q)\rangle|^2 + 2\text{Real}\left[\tilde{F}_\kappa(q)\tilde{F}_\kappa^*(q)\frac{\Omega_\kappa(q)}{\tilde{P}_{2\kappa}(q)[1 - \Omega_\kappa(q)]}\right] \tag{S.24}$$

where

$$\tilde{P}_\kappa(q) = \int dL'_\alpha g(L'_\alpha)\exp[i\kappa q(L'_\alpha - L')] \tag{S.25}$$

$$\tilde{F}_\kappa(q) = \int dL'_\alpha\, g(L'_\alpha)F(q, L'_\alpha)\exp[i\kappa q(L'_\alpha - L')] \tag{S.26}$$

$$\Omega_\kappa(q) = \tilde{P}_{2\kappa}(q)P(q) \tag{S.27}$$

and $\langle F(q)\rangle$ is the polydispersity averaging

$$\langle F(q)\rangle = \int dL'_\alpha g(L'_\alpha)F(q, L'_\alpha) \tag{S.28}$$

Figure 4e shows the best fit of the model to SAXS data, with its continuous electron density contrast profile shown in the inset of Fig 4f. Supplementary Figure 11a shows the structure factor

and Supplementary Figure 11b shows the L'-size distribution along the fiber direction with different draw ratio (L' is the length of crystal and transition region, Supplementary Figure 10) As draw ratio increases, the humps move to small q, i.e. larger length scales, and also become less significant (i.e. more structural disordering as shown in Supplementary Figure 11b for high draw ratios).

The fiber diameter can be statistically estimated by analyzing the SAXS intensity along the meridian direction. This intensity is given by two contributions as the new Guinier-Porod model described,[26]

$$I(q) = \frac{G}{q^s}\exp\left(\frac{-q^2R_g^2}{3-s}\right), for\ q \le q_1 \tag{S.29}$$

$$I(q) = \frac{D}{q^d}, for\ q \ge q_1 \tag{S.30}$$

where

$$q_1 = \frac{1}{R_g}\left[\frac{(d-s)(3-s)}{2}\right]^{1/2} \tag{S.31}$$

$$D = G\exp\left(\frac{-q_1^2R_g^2}{3-s}\right)q_1^{(d-s)} \tag{S.32}$$

Here, *G* and *D* are Guinier and Porod scale factors, respectively, d is the Porod exponent, s is the dimensionality parameter, $\mathbf{q_1}$ is the boundary wave vector transfer where Guinier and Porod contributions merge, and R_g is the radius of gyration. In this case, R_g is cross-sectional radius of gyration of the cylinders if we approximate the aligned fibers as a bundle of cylinders. The mean

radius of the fibers is thus given by $R = \sqrt{2}R_g$. Supplementary Figure 11c shows the fitting data by new Guinier-Porod model, and diameter R_g = 7.8 nm, R = 11.1 nm are obtained for 70× sample.

We were not able to reliably obtain the crystal and amorphous dimensions for the samples beyond 50× draw ratio, for example 70× and 90× samples because the bump features are much less obvious than low draw-ratio samples. Several possibilities could lead to the smearing of the scattering bumps in the SAXS data in those high draw ratio samples. At high draw ratio, the size uniformity of the crystallites and amorphous regions become more polydispersity and the transition region between the two phases also becomes less well-defined. This acts like the resolution smearing effect in the scattering and causes the oscillatory fringes in the SAXS curve less visible. Secondly, the scattering signal from highly stretched samples is significantly reduced due to less volume of the samples in the x-ray beam. In addition, the fibrous morphology has higher scattering background because of the form factor of the fiber shapes; and as the fiber gets smaller in dimensions, this structural background contribution moves closer to the q region where bumps are expected, leading to difficult background separation in the modeling. However, we would like to point out that the ratio of the amorphous region to the superlattice period length is in general agreement with the WAXS data, as we show in Fig. 4f.

WAXS and SAXS patterns: Supplementary Figure 12 and Supplementary Figure 13 show all the WAXS and SAXS patterns for the as-extruded (1×) and drawn films with different draw ratios, respectively.

Supplementary Note 5. Thermal conductivity model

Estimation of the amorphous phase thermal conductivity: we employed a one-dimensional heat transfer model to compute the film thermal conductivity, which depends on the thermal conductivities of the crystalline and amorphous regions, as well as the amorphous fraction (η) in one periodic unit (amorphous length / period length, Supplementary Figure 10). This model was built to unambiguously pinpoint the key role of the amorphous region, specifically how the thermal conductivity in the amorphous part changes at high draw ratios. The approximation that the thermal transport can be described by a 1D model is reasonable for high draw ratios, due to the fact that SEM observations and SAXS results (Fig. 1g-j, Supplementary Figure 3c, and Supplementary Figure 11c) suggest a very small fiber diameter of 10~50 nm. Such a 1D model contains a unit cell of total length L including a crystalline and an amorphous region. We note that the transition region as discussed before has been effectively included into the crystalline region (Supplementary Figure 14 and Supplementary Figure 10a). Adding all the heat resistances, the effective thermal conductivity of this 1D model is then given by $k = [(1-\eta)/k_c + \eta/k_a]^{-1}$, as already shown in the main text.

We first discuss the extraction of the structural parameters used in the equation. As mentioned above, the SAXS measurement characterizes periodicity of the lamellar superlattice. One can first obtain the period length from SAXS structure factor analysis. The lengths (and ratios) of different regions (amorphous / crystalline) can be further estimated by studying the electron density distribution. In Fig. 4f inset, we show the electron density profile with respect to the draw ratio measured on different samples. The middle region (Fig. 4f inset, electron density ~1) becomes larger and larger as draw ratio increases, and can be identified as the crystalline region, while the two sides where the electron density is close to zero are the amorphous regions. We take the

amorphous region as the part where the electron density is less than 0.05 (corresponding to a length L_A). The remaining part ($L - L_A$) is taken as the effective crystallite size L_C. The ratio between the amorphous region and the total length L_A/L gives the amorphous fraction η (Fig. 4f, circles).

Due to the uncertainties involved in estimating the lengths from the SAXS measurement data, we do not directly use the experimental data points for η and L_C in the thermal model. Instead, we fit these values by a simple functional form $\eta \ (or\ L_c) = C_1 * n^{C_2}$($n$ being the draw ratio), which should generally capture the trend of the structural parameters as the draw ratio. For the amorphous fraction, the fitting to the experimental values yields $C_1 = 0.6$ and $C_2 = -0.43$. To take into account the large uncertainty associated with these values, 40% variations have been added to them, which translate into the upper and lower bounds for the estimated η as shown in Fig. 4f (shaded region). Similarly, for the crystallite size, we obtained $C_1 = 7\ nm$ and $C_2 = 0.28$, with a 20% variation considered that leads to a range for the estimated crystallite size as the draw ratio, shown in Supplementary Figure 15.

Thermal conductivity of the crystalline region depends on both the crystallite width and length. We used the chain length-dependent thermal conductivity of a single polyethylene chain calculated using the first principles method by Huang as an approximation of the crystalline region thermal conductivity[27]. Although the same paper also gave lateral size dependent thermal conductivity of nanocrystals, it assumed diffuse phonon scattering at the boundaries which may be too severe an approximation because (1) the weak van der Waals interaction between the crystalline and amorphous regions, and (2) the transition from the crystalline to the amorphous region is gradual as from the SAXS data, suggesting some molecules may extend from the crystalline to the amorphous region. The simulated length-dependent thermal conductivity data for the 1D chain are then fitted with a quadratic function, as shown in Supplementary Figure 15a (the

functional form is $k_c[\mathrm{W\,m^{-1}\,K^{-1}}] = -23915 \cdot l^2 + 3446.5 \cdot l + 10.398$, where l is the chain length). We then take the measured crystallite size at different draw ratios (Supplementary Figure 15b) and plug into this quadratic function. This yields the crystalline thermal conductivity as a function of the draw ratio (Supplementary Figure 15c) to be used in our thermal modeling. Our following sensitivity analysis on the crystalline thermal conductivity shows that the cited amorphous region thermal conductivity is a conservative estimate. In plotting Fig. 3b, the shaded region is obtained by fitting the measured total thermal conductivity with a straight line ($3.8 + 0.5 \times n$, W m^{-1} K^{-1}). The upper and lower bounds originate from the uncertainties we considered in the estimation of crystallite sizes as well as the amorphous fractions, as mentioned above.

Finally, we mention that though one-dimensional heat conduction model has been adopted, there is still possibility that the heat may flow across different polyethylene nanofibers. Here we justify our 1D model by showing that the thermal resistance of this curved heat flow is too large to explain our experimentally measured thermal conductivity.

In Supplementary Figure 14c, we show the schematic of the heat flow along a curved path across two different nanofibers. We will focus on one single fiber (fiber A), and estimate the thermal resistance of heat flow from fiber A to the surrounding fibers and then back to fiber A. We consider path starting at red dashed line and ends at blue dashed line, with a total length corresponding to one repeated unit.

For estimation, we take geometry data for the 50× drawn sample (crystalline thermal conductivity k_c ~ 70 W m^{-1} K^{-1}, repeated unit length L_{tot} ~ 22 nm, crystalline domain length L_c ~ 20 nm) and take the nanofiber diameter D to be 10 nm. It is important to note that the nanofibers assemble into bundles mostly via van der Waals interactions, which creates large interfacial

thermal resistance. We estimate the interfacial thermal conductance h to be around $3 \times 10^7\ \mathrm{W\,m^{-2}\,K^{-1}}$ based on literature data [28] for clean interface with van der Waals bonding. Because the interface in our case is not atomically flat, the actual thermal conductance can be even lower. We also neglect the thermal resistance in the surrounding fibers, effectively treating them to be at uniform temperatures. These simplifications represent the worst scenario which will only underestimate the total thermal resistance.

We estimate the thermal resistance from fiber A to surrounding fibers using a heat transfer fin model assuming that the fiber exchanges heat with surroundings with a heat transfer coefficient h. This model treats all neighboring fibers as a uniform environment, representing the worst case of multiple fibers closely packed with an infinite thermal conductivity. Based on the parameters we provided ($h = 3 \times 10^7\ \mathrm{W\,m^{-2}\,K^{-1}}$, $k_c = 70\ \mathrm{W\,m^{-1}\,K^{-1}}$, and $D = 10$ nm), the Biot number ($\mathrm{Bi} = h \cdot D/k_c = 0.0043$) is much less than 1 and the fin approximation is thus justified. The thermal resistance between the starting point in fiber A and the surrounding is approximately

$$R_{fin,eff} = \frac{1}{\sqrt{k_c A h P} \cdot \tanh\left(m L_c/2\right)} \tag{S.33}$$

where $A = \pi D^2/4$, $P = \pi D$, and $m = \sqrt{\frac{hP}{k_c A}}$ is the fin parameter. This leads to $R_{fin,eff} \sim 1.04 \times 10^8\ \mathrm{K\,W^{-1}}$. As the curved path involves passing interfaces twice, the total thermal resistance will be $2R_{fin,eff} \sim 2.1 \times 10^8\ \mathrm{K\,W^{-1}}$.

If the curved path were the dominant heat conduction channel, the corresponding thermal conductivity based on the area and repeated unit length of the nanofiber should match our experimental measurement. However, the corresponding thermal conductivity for the heat flow along the curved path is

$$k_{tot} = \frac{L_{tot}}{A}\frac{1}{2R_{fin,eff}} = 1.3\ \mathrm{W\ m^{-1}\ K^{-1}} \tag{S.34}$$

which is much smaller than the measured thermal conductivity (~ 30 W m^{-1} K^{-1} at 50×). The above analysis depends on the interfacial thermal conductance h, for which we have assumed a small value (3×10^{7} W m^{-2} K^{-1}). Even if h takes a larger value, e.g. h $=1\times10^{8}$ W m^{-2} K^{-1}, the corresponding thermal conductivity along the curved path will only see a modest increase (k_{tot} ~ 4.3 W m^{-1} K^{-1}), which is still far from being able to explain our data. Considering all these, we conclude that the curved path has negligible contribution to the total heat conduction.

Sensitivity analysis: here we provide a sensitivity analysis for the relevant parameters (crystalline state thermal conductivity k_c, and amorphous fraction η), to show that our conclusion of amorphous phase developing a large thermal conductivity is unaltered by the uncertainties involved in determining the structural parameters. We use the 50× drawn sample as an example. We first evaluate the sensitivity of the estimated amorphous thermal conductivity k_a to k_c. In Supplementary Figure 16a, we plot the variation of k_a with different values of k_c. As stated above, the value of k_c is determined by combining our measured crystallite size and the literature reported size-dependent single chain polyethylene thermal conductivity. At 50× we obtained k_c ~ 70 W m^{-1} K^{-1}, corresponding to k_a ~ 5 W m^{-1} K^{-1}. Although there are uncertainties involved in determining the crystallite size, it is clear from Supplementary Figure 16a that for any value of k_c, k_a is always larger than ~ 4 W m^{-1} K^{-1}, a value significantly larger than its bulk case. This is simply because, when there is certain fraction of the amorphous phase, the amorphous thermal conductivity has to be sufficiently large to ensure good thermal conductance. In other words, the uncertainties in k_c do not affect our conclusion.

Of course, if the fraction of the amorphous region is zero, then it is unnecessary to invoke a large amorphous phase thermal conductivity to explain our measured total thermal conductivity because a large crystalline thermal conductivity itself suffices. However, we believe our structural characterization has provided sufficient evidence to show that the fraction of the amorphous region is not zero (Fig. 4, Supplementary Figure 12 and Supplementary Figure 13). As stated above, the amorphous fraction is estimated from SAXS measurement. For the 50× drawn sample, the sensitivity of k_a to the amorphous fraction parameter (η) is shown in Supplementary Figure 16b. At 50×, our estimated amorphous fraction is η ~ 11% (Fig. 4f), corresponding to k_a ~ 5 W m^{-1} K^{-1}. This amorphous fraction is consistent with the crystallinity measured for 50× drawn sample which is ~ 84% (Fig. 4d). We have estimated the amorphous fraction based on SAXS rather than crystallinity because we believe SAXS provides a more quantitative measure. Nonetheless, if we instead inferred the amorphous fraction based on crystallinity to give η ~ 16%, k_a will be even higher (~7 W m^{-1} K^{-1}, Supplementary Figure 16b). The uncertainty involved in determining the amorphous fraction has been taken into account, as shown by the shaded area in Fig. 4f. In case that the actual amorphous fraction is 50% less than the current value (η ~ 6%), k_a will be ~ 2.5 W m^{-1} K^{-1} (Supplementary Figure 16b), still significantly larger than its bulk situation. Therefore, we have shown that within the possible range of the amorphous fraction η, k_a could vary but is always significantly larger than its bulk value.

Furthermore, as we mentioned in the manuscript, reference[27] also gave simulated thermal conductivity of different polyethylene crystallites assuming diffuse boundary scattering. Under this assumption, the thermal conductivity of a 30 nm crystallite at room temperature is ~ 40 W m^{-1} K^{-1}, which is even smaller than the total thermal conductivity we measured. As explained in the manuscript, we believe that the diffuse boundary scattering maybe too conservative since the

interface between the crystalline and amorphous regions is of van der Waals in nature and also some molecules may extend from the crystalline to the amorphous regions. A smaller thermal conductivity of the crystalline region only leads to a larger amorphous region thermal conductivity. Hence, we believe that the stated thermal conductivity of the amorphous region represents conservative lower limits.

**Draw ratio**	**Length (mm)**	**Cross-sectional area (mm^2)**	Thermal conductance ($W K^{-1}$)
1	1.3	0.6876	2.19E-04
2.5	1	0.10395	5.45E-04
5	1.1	0.06435	3.73E-04
10	2	0.055858	3.34E-04
20	1.5	0.050818	5.08E-04
30	2	0.028584	3.85E-04
40	2.2	0.043407	4.65E-04
50	2.25	0.021630	3.21E-04
60	2.2	0.016458	2.53E-04
70	1.65	0.0083974	2.32E-04
80	1.15	0.010078	4.68E-04
90	1.1	0.0075624	3.81E-04
100	0.65	0.0068800	6.02E-04
110	1	0.0052500	3.58E-04

Supplementary Table 1. Measured sample dimensions. Polymer film dimensions at various draw ratios.

**Standard sample**	**Length (mm)**	**Cross-sectional area (mm^2)**	Thermal conductance ($W K^{-1}$)
Dyneema	1.36	0.06810	1.21E-03
Zylon	1.21	0.07000	1.31E-03
S. Steel 304	3.2	0.4725	2.24 E-03
Sn	5.55	0.03425	4.48 E-04
Sn	3.80	0.02500	5.08E-04
Sn	2.55	0.01300	3.85E-04
Al	5.79	0.01285	4.92E-04
Al	4.30	0.01285	6.57E-04

Supplementary Table 2. Measured standard sample dimensions. These include 304-stainless steel foils[6], Zylon fibres[2], Dyneema fibres[2], Sn[7] and Al[7] films.

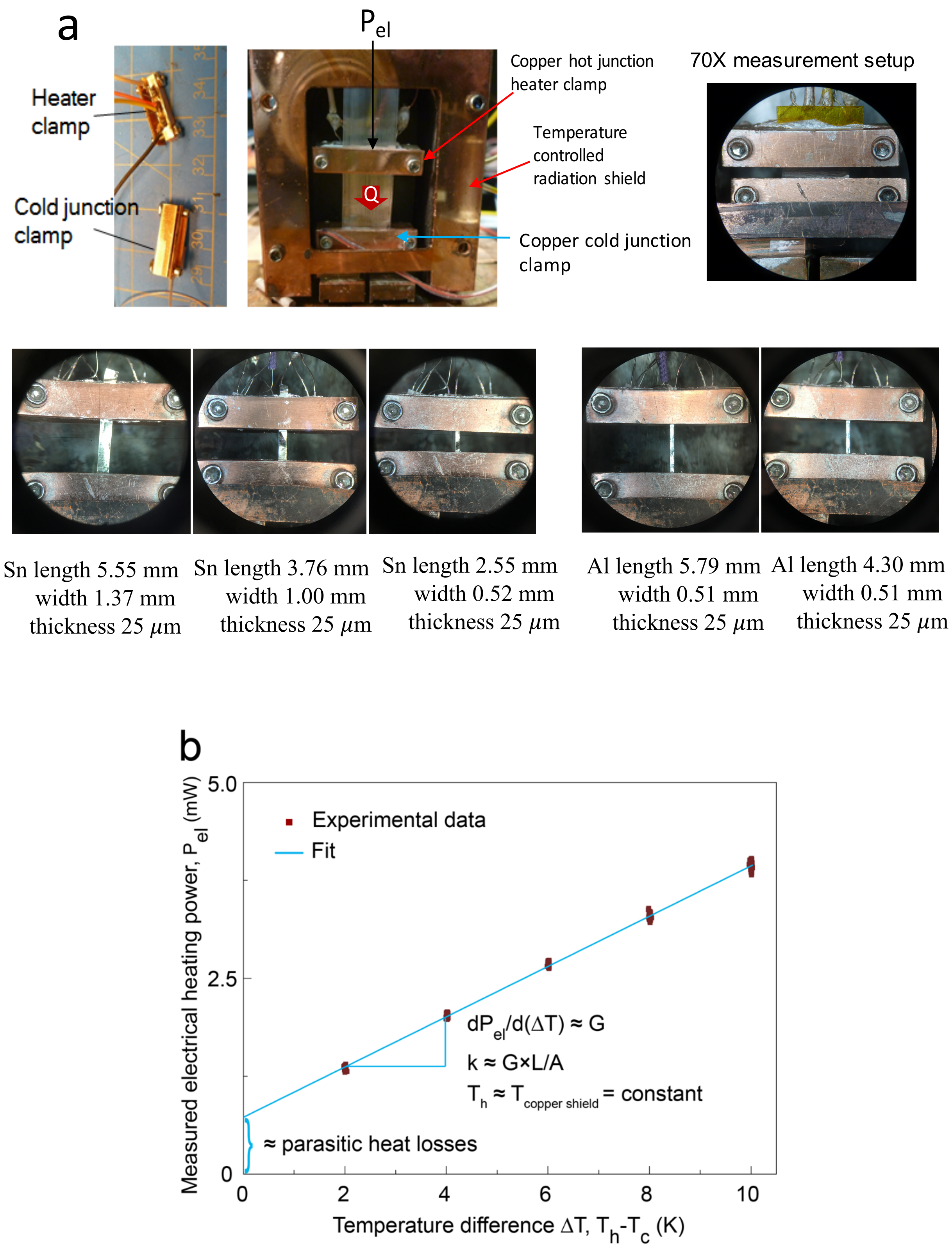
a
Pel
Heater clamp
Cold junction clamp
Q
Copper hot junction heater clamp
Temperature controlled radiation shield
Copper cold junction clamp
70X measurement setup
Sn length 5.55 mm width 1.37 mm thickness 25 μm
Sn length 3.76 mm width 1.00 mm thickness 25 μm
Sn length 2.55 mm width 0.52 mm thickness 25 μm
Al length 5.79 mm width 0.51 mm thickness 25 μm
Al length 4.30 mm width 0.51 mm thickness 25 μm
b
Experimental data
Fit
dPel/d(ΔT) ≈ G
k ≈ G×L/A
Th ≈ Tcopper shield = constant
≈ parasitic heat losses
Measured electrical heating power, Pel (mW)
Temperature difference ΔT, Th-Tc (K)
0
2.5
5.0
0
2
4
6
8
10

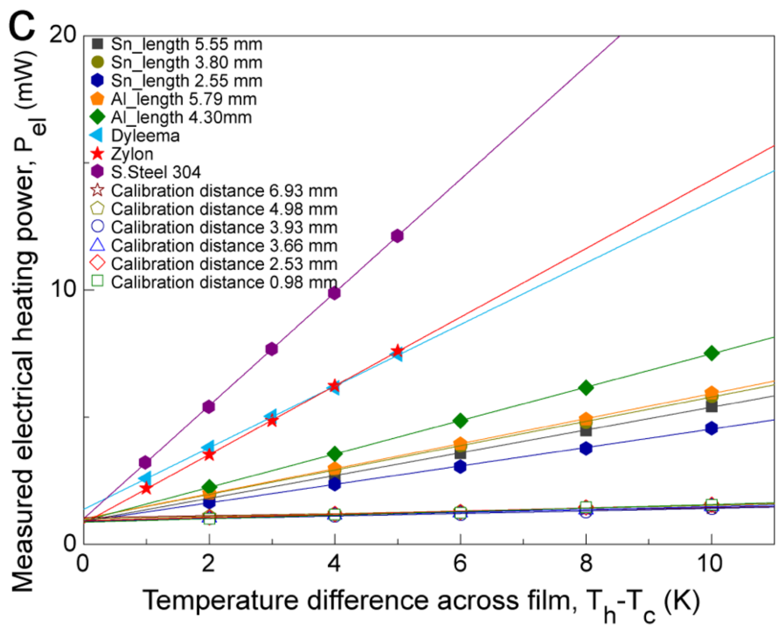
c
Sn_length 5.55 mm
Sn_length 3.80 mm
Sn_length 2.55 mm
Al_length 5.79 mm
Al_length 4.30mm
Dyleema
Zylon
S.Steel 304
Calibration distance 6.93 mm
Calibration distance 4.98 mm
Calibration distance 3.93 mm
Calibration distance 3.66 mm
Calibration distance 2.53 mm
Calibration distance 0.98 mm
Measured electrical heating power, P_el (mW)
Temperature difference across film, T_h-T_c (K)
0
2
4
6
8
10
20

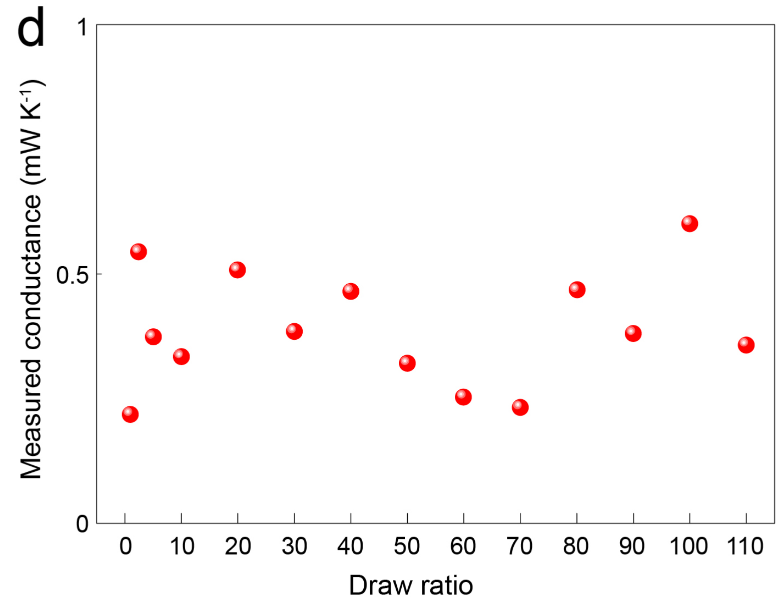
d
Measured conductance (mW K-1)
Draw ratio
0
0.5
1
0
10
20
30
40
50
60
70
80
90
100
110

e

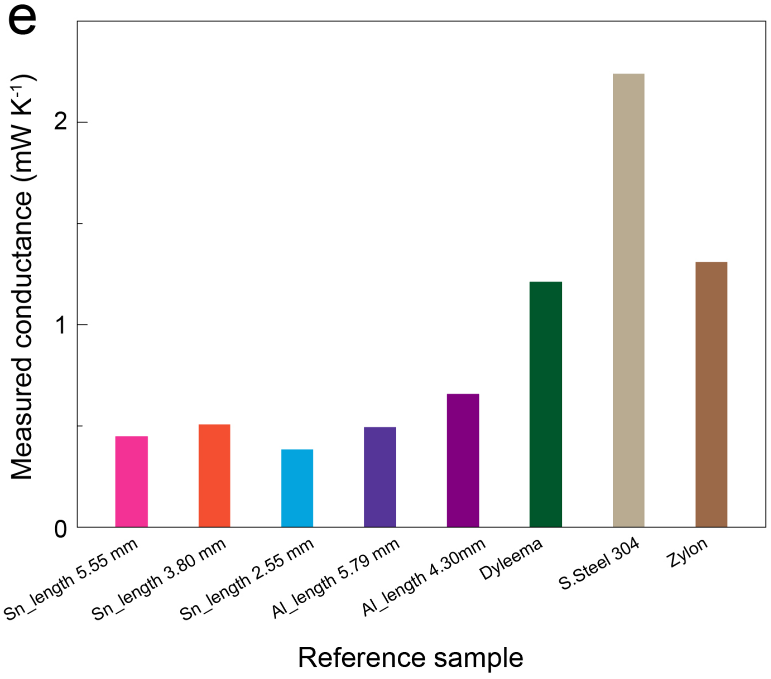

Measured conductance (mW K-1)
0
1
2
Sn_length 5.55 mm
Sn_length 3.80 mm
Sn_length 2.55 mm
Al_length 5.79 mm
Al_length 4.30mm
Dyleema
S.Steel 304
Zylon
Reference sample

f

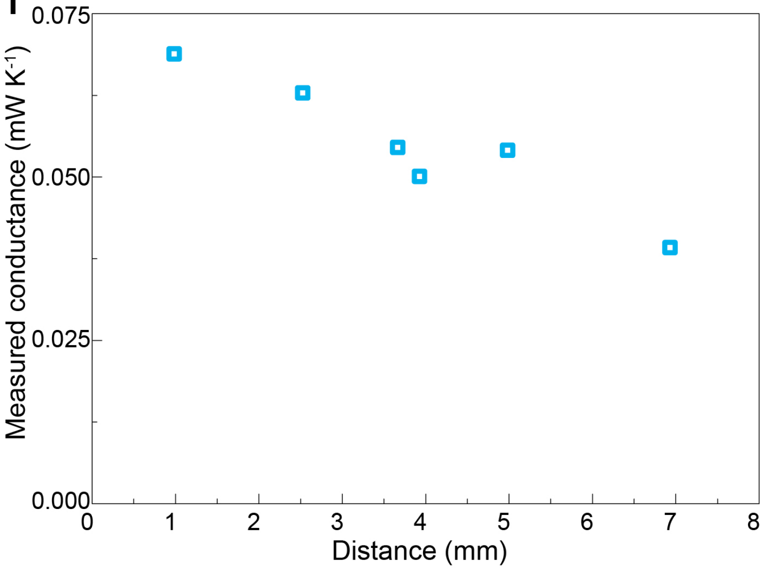

Measured conductance (mW K-1)
0.000
0.025
0.050
0.075
0
1
2
3
4
5
6
7
8
Distance (mm)

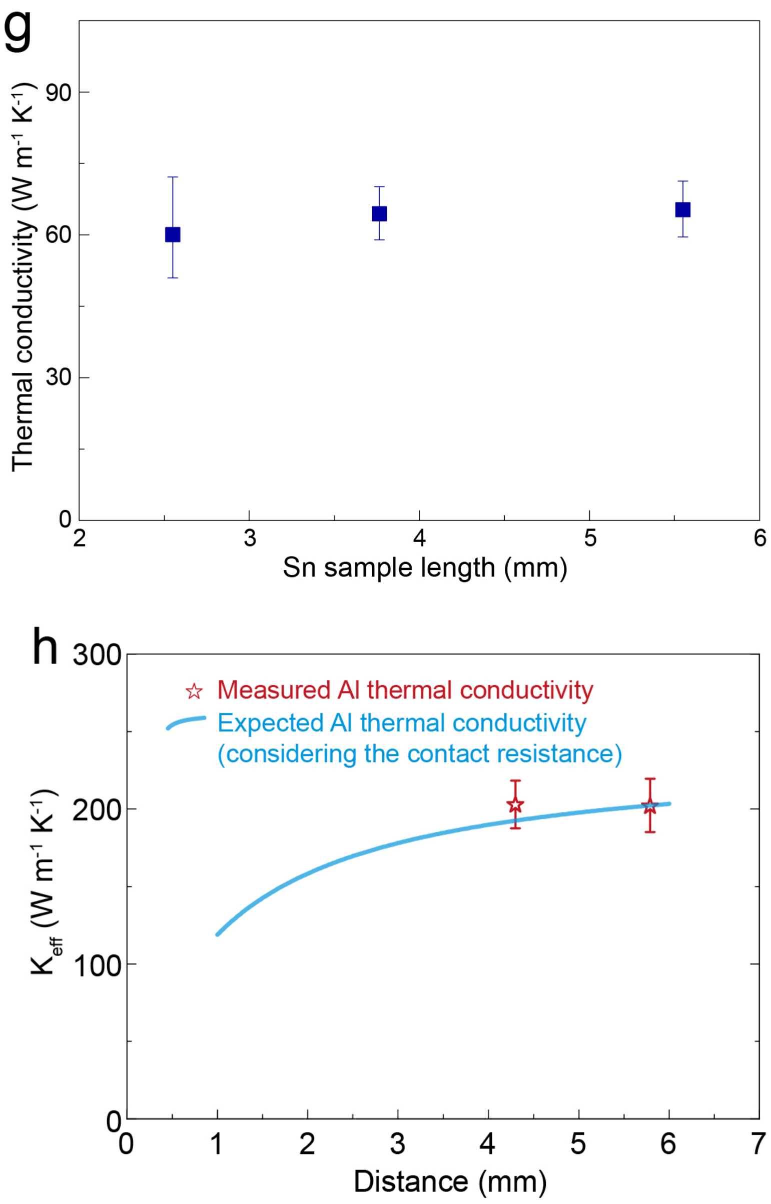
g
Thermal conductivity (W m-1 K-1)
90
60
30
0
2
3
4
5
6
Sn sample length (mm)
h
300
200
100
0
Measured Al thermal conductivity
Expected Al thermal conductivity
(considering the contact resistance)
Keff (W m-1 K-1)
0
1
2
3
4
5
6
7
Distance (mm)

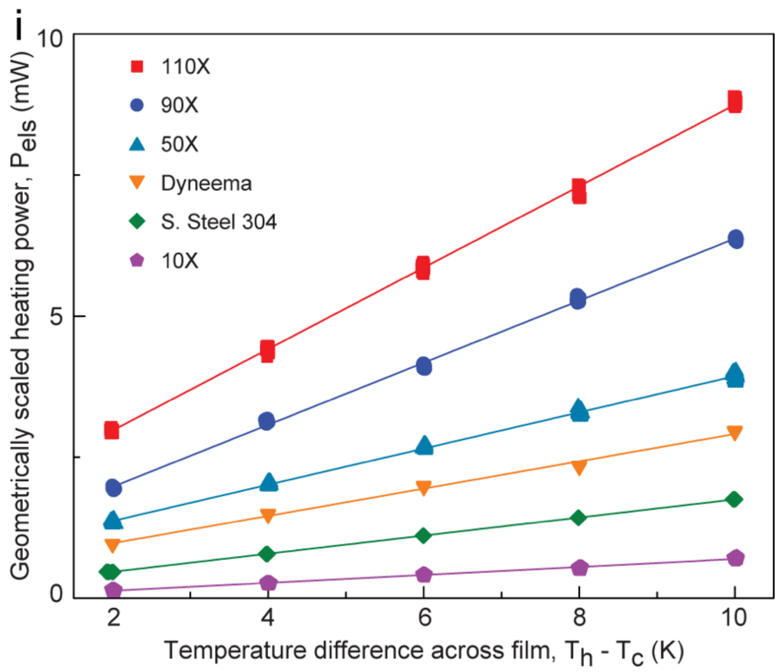

Supplementary Figure 1. Differential steady-state method to measure thermal conductivity. **a** Experimental setup photos showing a plastic film suspended between the hot and cold copper clamps, as well as the temperature-controlled copper radiation shield. Representative setup for 70× drawn film, reference samples (Al[7] and Sn[7] films) with different geometries. Detailed sample geometry is shown in Supplementary Table 1. **b** Representative data from measurement of a 50× film illustrating the differential nature of the method. Electrical heating power (P_{el}) is measured at a series of temperature differences. The temperature difference is varied by changing the cold side temperature while maintaining the heater temperature constant. The linear fit slope corresponds to the thermal conductance, $G = kA/L$, of the sample. **c** Directly measured electrical heating power (P_{el}) for all reference samples and calibration tests without mounting samples. We measured thermal conductivities of ~15.3 (+1.1, −0.6) W m^{-1} K^{-1}, ~22.6 (+5.0, −4.5) W m^{-1} K^{-1}, ~23.6 (+4.4, −3) W m^{-1} K^{-1}, ~64.4 (+5.7, −5.4) W m^{-1} K^{-1}, and ~202.7 (+15.62, −15.06) W m^{-1} K^{-1} for 304-stainless steel foils[6], Zylon fibres[2], Dyneema fibres[2], Sn[7] and Al[7] films, respectively. **d** Measured thermal conductance of all the polyethylene films. **e** Measured thermal conductance of all the reference samples: Dyneema[2], Zylon[2], stainless steel[6], Sn[7] and Al[7] films. **f** Measured thermal conductance of calibration tests. **g** Measured thermal conductivity for Sn films with different geometry (Supplementary Table 2). The error bars take into account the uncertainties in the measurement of the Sn film geometry, the uncertainty in the estimation of the radiation contribution (Sn emissivity at room temperature takes 0.04 with 50% uncertainty considered) and the uncertainty in the thermal shunting measurement. **h** Measured thermal conductivity for Al films with different geometry (Supplementary Table 2). The error bars represents the uncertainties in the measurement of the Al film geometry, the uncertainty in the estimation of the radiation contribution (Al emissivity at room temperature takes 0.07 respectively with 50% uncertainty considered) and the uncertainty in the thermal shunting measurement. Detailed analysis of geometric uncertainties is discussed in Supplementary 2. Blue curve represents expected thermal conductivity of aluminum from measurement with respect to the sample length, considering the contact resistance, following the analysis given in Supplementary Equation 5 and Supplementary

Equation 6 but replacing k_{CP} by $t/2R_C$, where R_C is the contact resistance. An R_C value of 4.2×10^{-5} m^2 K W^{-1} gives thermal conductivities matching our experiments, and this R_C value corresponds to a 42 μm layer thick silver epoxy paste if the uncured silver epoxy has a thermal conductivity of 1 W m^{-1} K^{-1} and it is reasonable. The cross-sectional area takes the sample geometry (width is 0.51mm, and thickness is 25μm). The contact resistance value is within reasonable range for interfaces between metals, explaining the lower value of our measured thermal conductivity for the highly conductive reference samples. Compared with reference value for Al thermal conductivity[7], we always underestimate the Al thermal conductivity by our steady-state method. **i** Geometrically scaled electrical heating power (P_{els}) as a function of the temperature difference (T_h-T_c) across films. Representative data (10×, 90×, 110×, Dyneema and S. Steel 304) are scaled to the geometry of a 50Å~ film ($P_{els} = P_{el} \cdot (A/L)_{50\times} / (A/L)$). A larger slope indicates a higher thermal conductivity.

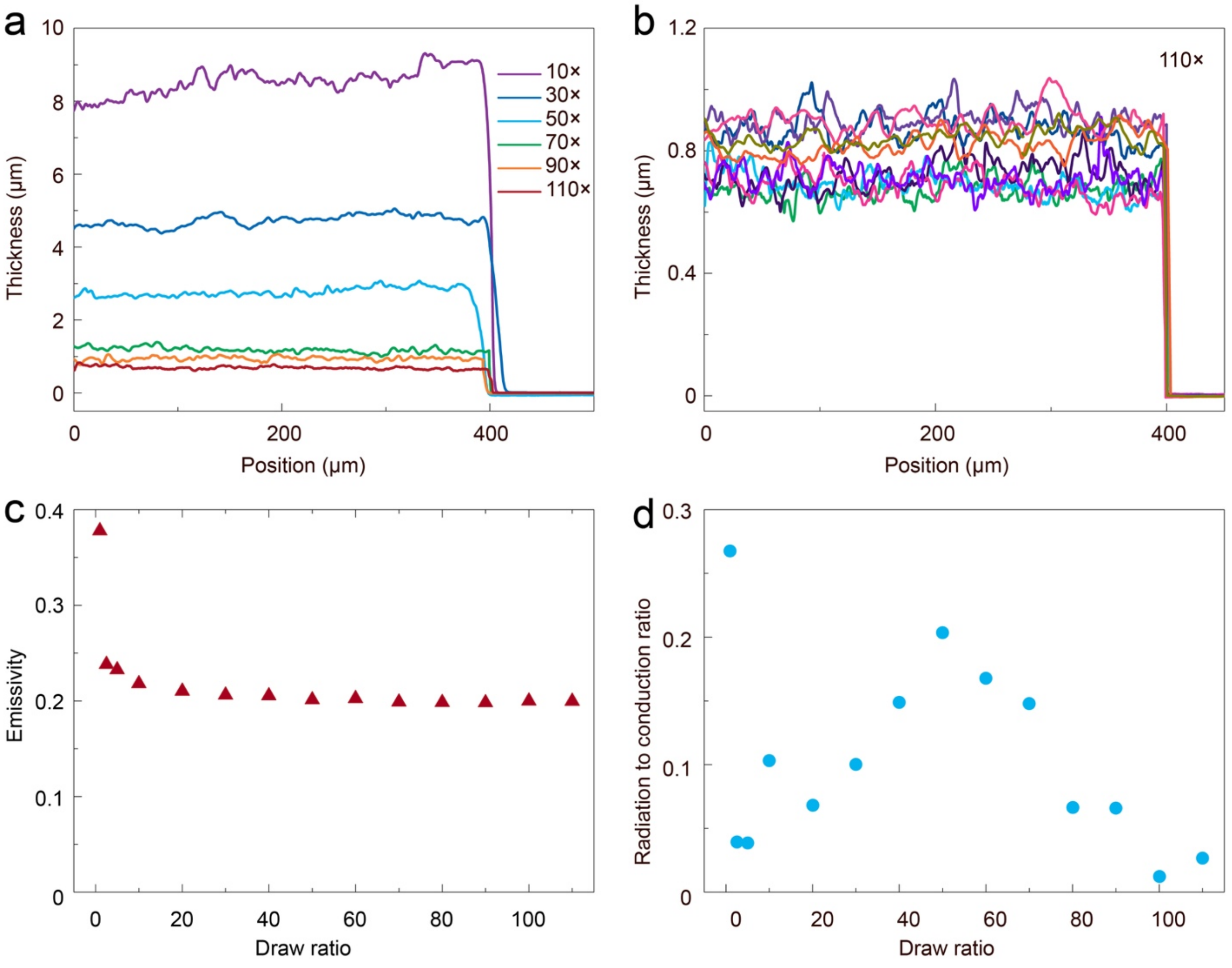

Supplementary Figure 2. Polyethylene film thickness and radiation error analysis. a Drawn polyethylene film thickness is in the range of ~ 1-8 μm, as measured by a stylus profilometer. **b** Representative profiles for 110× film at 10 different locations along the sample. **c** Computed emittance for various polyethylene films at 298 K. **d** Radiation error as a function of draw ratio. In general, radiation errors are less than 25%, and for the higher draw ratios, less than 10%.

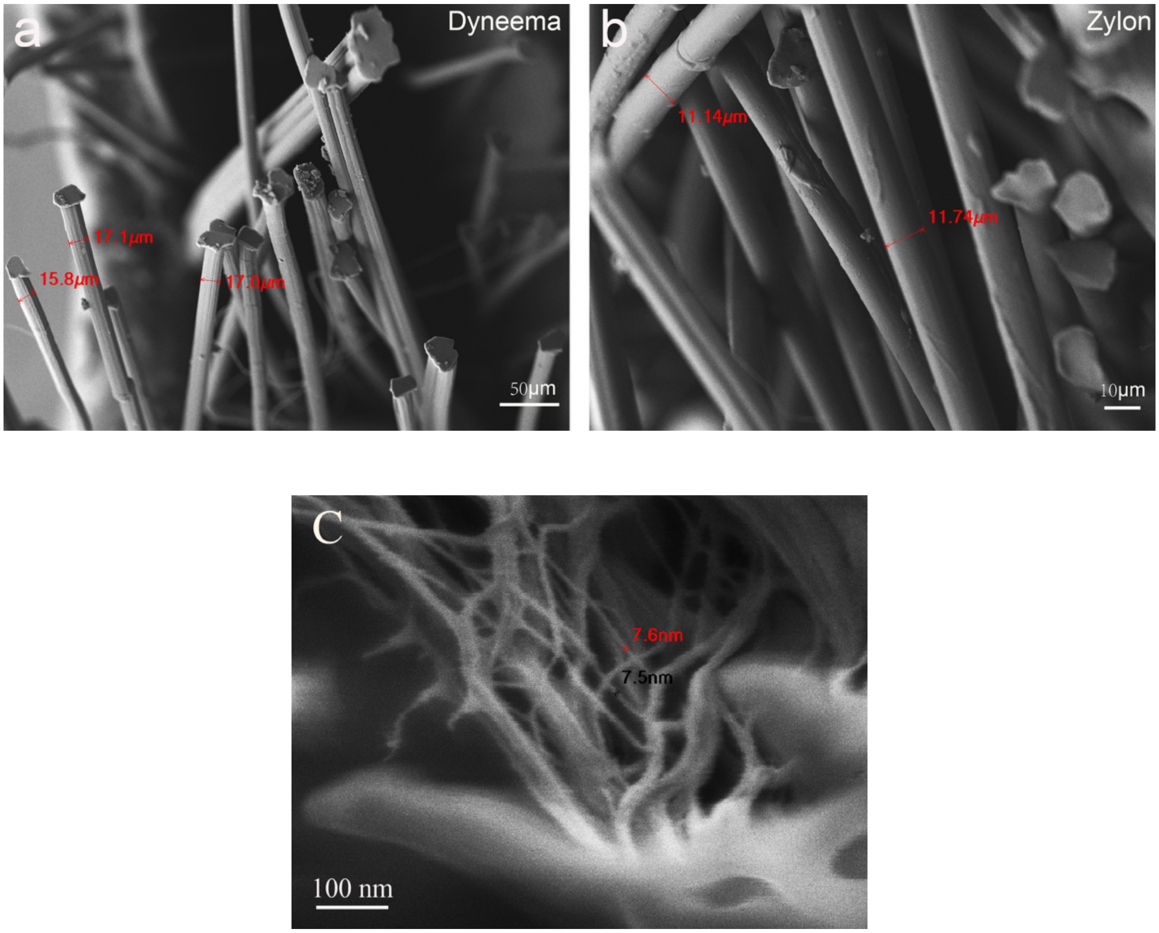

Supplementary Figure 3. SEM images. a Average Dyneema fiber diameter is ~17 μm. Scale bar indicates 50 μm. **b** Zylon fiber diameter is ~11.7 μm. Scale bar indicates 10 μm. **c** Images of a torn 70× polyethylene film in this work, the interior nanofiber diameters is ~ 8 nanometers. Scale bar indicates 100 nm.

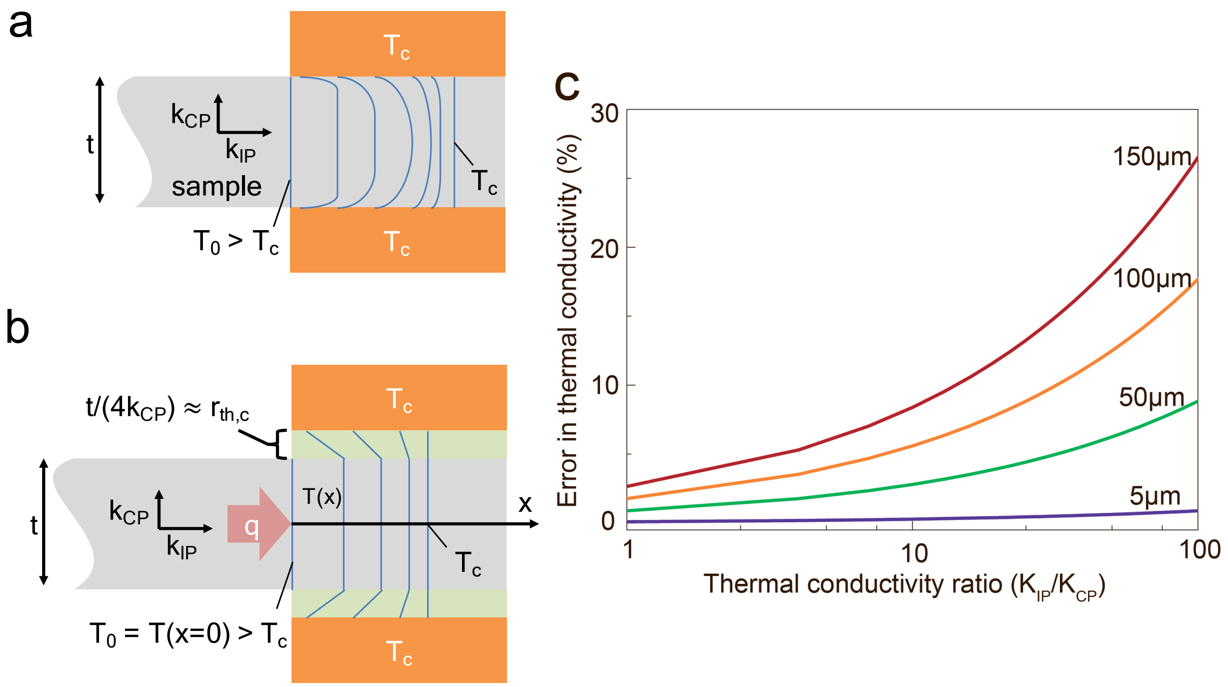

Supplementary Figure 4. Effect of anisotropic thermal conductivity of drawn polyethylene films. a Temperature profile along the sample thickness is non-uniform in the clamped region, and temperature on the left side of the clamp is inevitably higher than that of the clamp. **b** Simplified model to estimate effect of the cross-plane (2D) fin resistance on the thermal conductivity results. Cross-plane thermal resistance of the sample is lumped into an effective thermal contact resistance $r_{th,c} \approx t/4k_{cp}$. **c** Modeling results for a 4-mm long sample show a small error for thin film thicknesses (< 5 µm), even when the thermal conductivity anisotropy ratio is more than 200. The error associated with anisotropy dramatically increases for thicker films. Therefore, to minimize the fin resistance effect on the thermal conductivity measurement the sample should be long and thin.

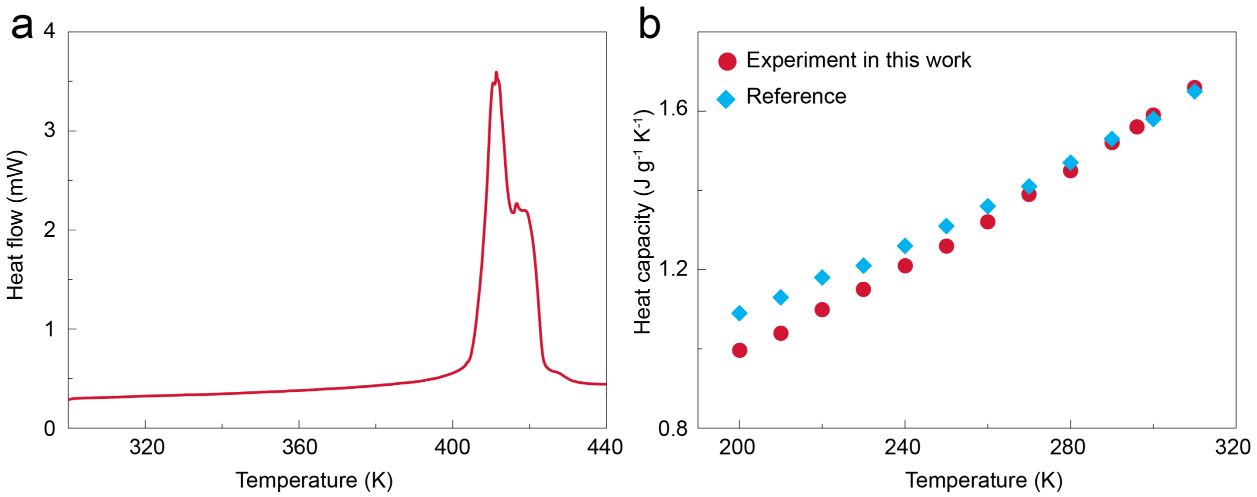

Supplementary Figure 5. Melting temperature and specific heat capacity. a Melting temperature of the 50× films is ~140 °C, as indicated by the peak in heat flow using differential scanning calorimetry. **b** Specific heat capacity of 50× films as a function of temperature. Measured specific heat of the 50× films agree well with previously reported values[14], especially near room temperature. The average data from three temperature cycles are plotted.

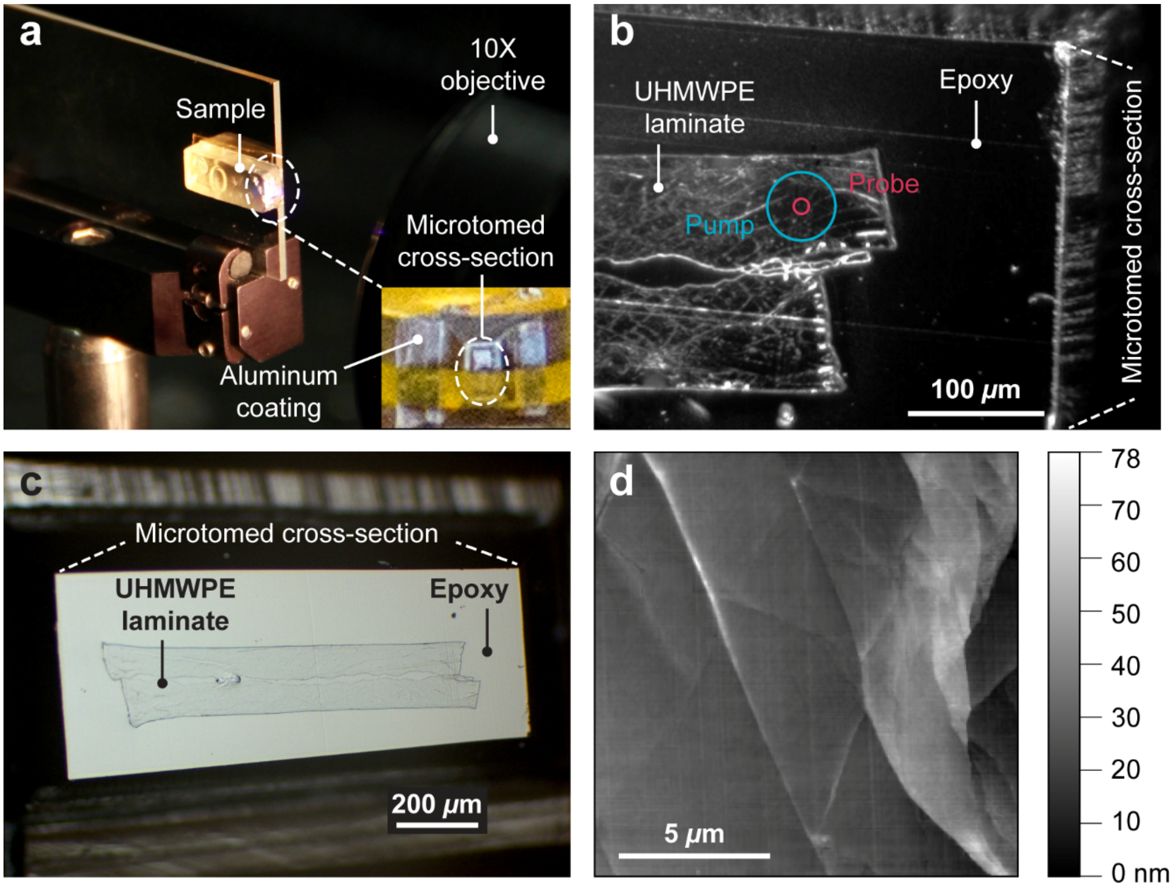

Supplementary Figure 6. Images across multiple length scales of a TDTR sample. The sample features a UHMWPE laminate embedded in an epoxy matrix, and was carefully cut with a microtome at room temperature in order to reveal a flat and smooth cross-section for TDTR measurement. **a** Photo of the sample mounted in front of a long-working-distance 10× microscope objective. Inset is a zoom-in view of the microtomed cross-section partially coated with an 88 nm-thick aluminum layer. **b** Dark-field optical micrograph of the sample cross-section obtained during a TDTR measurement. The UHMWPE laminate cross-section is ~1 mm × 150 μm and consists of 100 layers of as-drawn 50× films hot pressed together. It separated into two halves during sample preparation. Smooth and dark regions generally indicate good sample surface quality. The blue and red circles show the pump (53 μm in diameter) and probe (11 μm) spots, respectively. Scale bar indicates 100 μm. **c** Bright-field optical micrograph of the cross-section prior to e-beam evaporation of aluminum. Scale bar indicates 200 μm. **d** AFM image of the UHMWPE laminate cross-section. The root-mean-square surface roughness is ~10 nm. Scale bar indicates 5 μm.

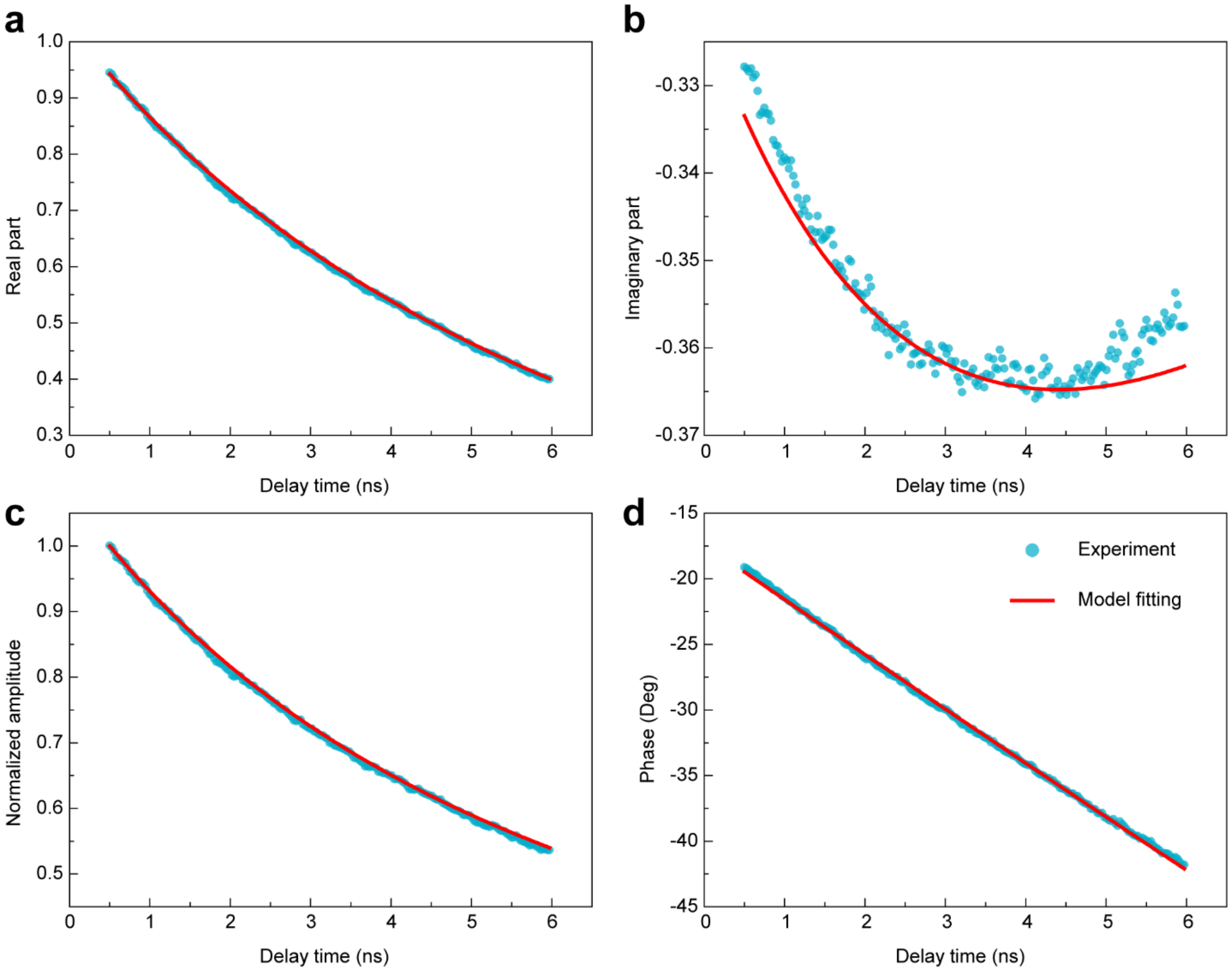

Supplementary Figure 7. Measured and fitted complex thermoreflectance signals. The measured data is the average of 10 individual runs using a modulation frequency of 6 MHz. **a-b** Real and imaginary parts of the complex signal, respectively. **c-d** Amplitude (normalized) and phase representation of the same signal, respectively. Phase fitting was performed to obtain the sample thermal conductivity together with the aluminum/sample interface thermal conductance, which were subsequently used to compute all the red curves. The reliability of the experimental results is demonstrated by the fact that fitting to phase alone leads to excellent agreement between modeled and measured data in all four panels. As expected, fitting of the amplitude yields equally good results (see Fig. 2d).

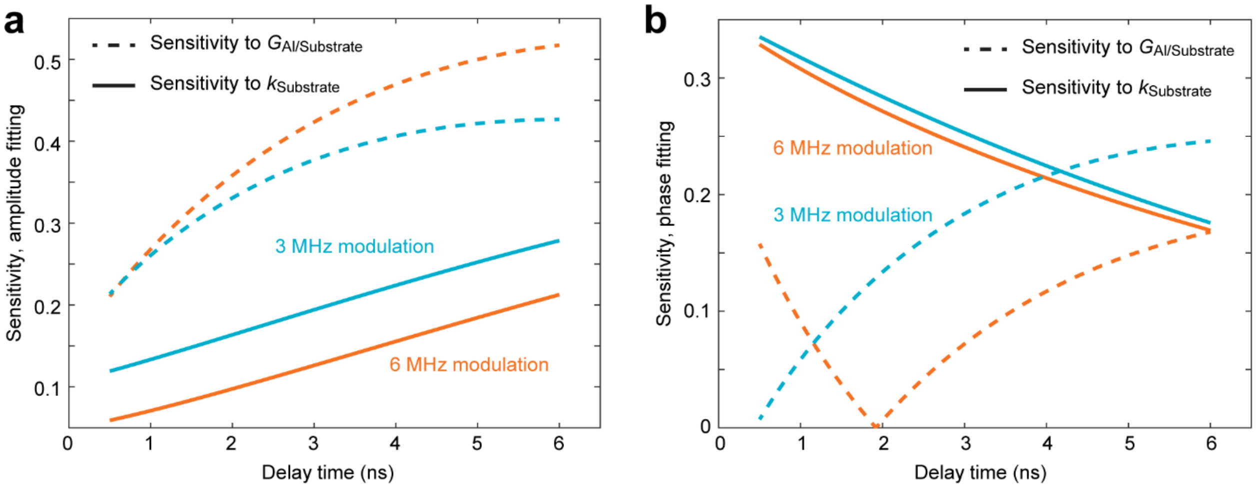

Supplementary Figure 8. Sensitivity analysis of the TDTR experiment. a Sensitivity[9,10] of the amplitude of the complex thermoreflectance signal to the aluminum/substrate (UHMWPE laminate) interface thermal conductance ($G_{\mathrm{Al/Substrate}}$) and substrate thermal conductivity ($k_{\mathrm{Substrate}}$). **b** Sensitivity when fitting to the phase of the thermoreflectance signal. Although amplitude fitting and phase fitting offer different relative sensitivity to the substrate conductivity and the interface conductance, both are sufficiently sensitive considering the relatively small experimental noise and more importantly the excellent agreement between results from phase and amplitude fitting.

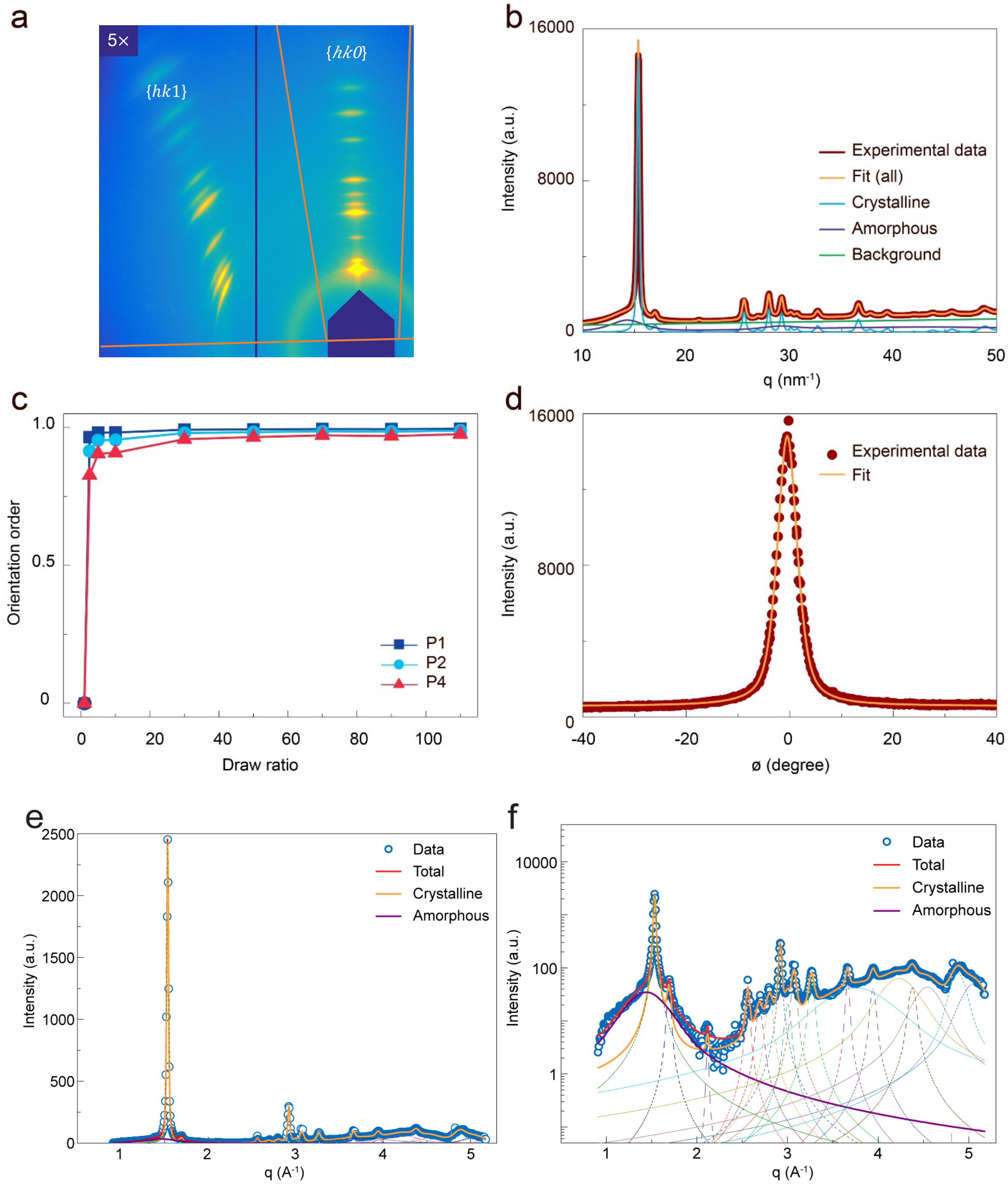

Supplementary Figure 9. Effective crystallinity and crystallite orientation analysis from WAXS. a WAXS pattern of the 5× films, the limited active area of the detector captures only a little over a quarter of the entire WAXS pattern, i.e. the {*hk0*} and {*hk1*} Bragg groups. **b** 1D profile with best fits of the crystalline, amorphous and a linear background peaks by Voigt model. **c** Orientation order parameters of the films at different draw ratios. **d** The intensity profile from the (*200*) peak as a function of the azimuthal angle ∅. The peak is first fitted to a Voigt function, which is then used for the calculation of the order parameters. **e** The crystallinity analysis for the drawn polyethylene at 110× draw ratio (linear scale). **f** The crystallinity analysis for the drawn

polyethylene at 110× draw ratio (Log scale). Log scale is used to enhance the significance of amorphous contribution to the crystallinity determination.

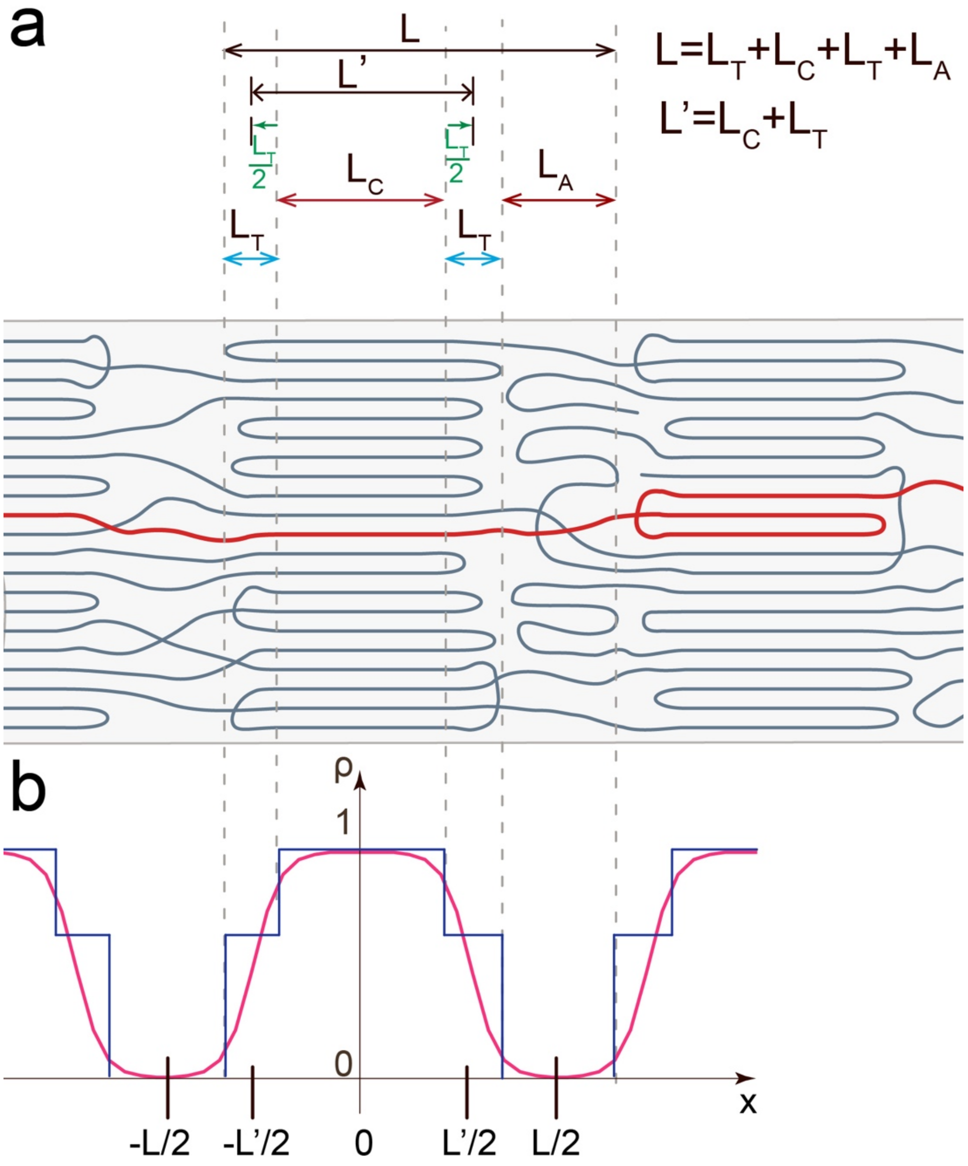

Supplementary Figure 10. Schematic of the lamellar superlattice structure and electron density profile. a Each repeating unit of the lamellar superlattice is of a total length L, which includes three phases: a crystal phase of length L_C, an amorphous phase of length L_A, and two transition layers of length L_T in between to represent an electron density change from crystalline to amorphous. **b** Step-like three phase model describing the electron density profile of the unit is simplified by a two-phase model with a continuous density profile. In the two-phase model, the transition is modeled by an error function profile (whose derivative is a Gaussian of standard deviation of σ).

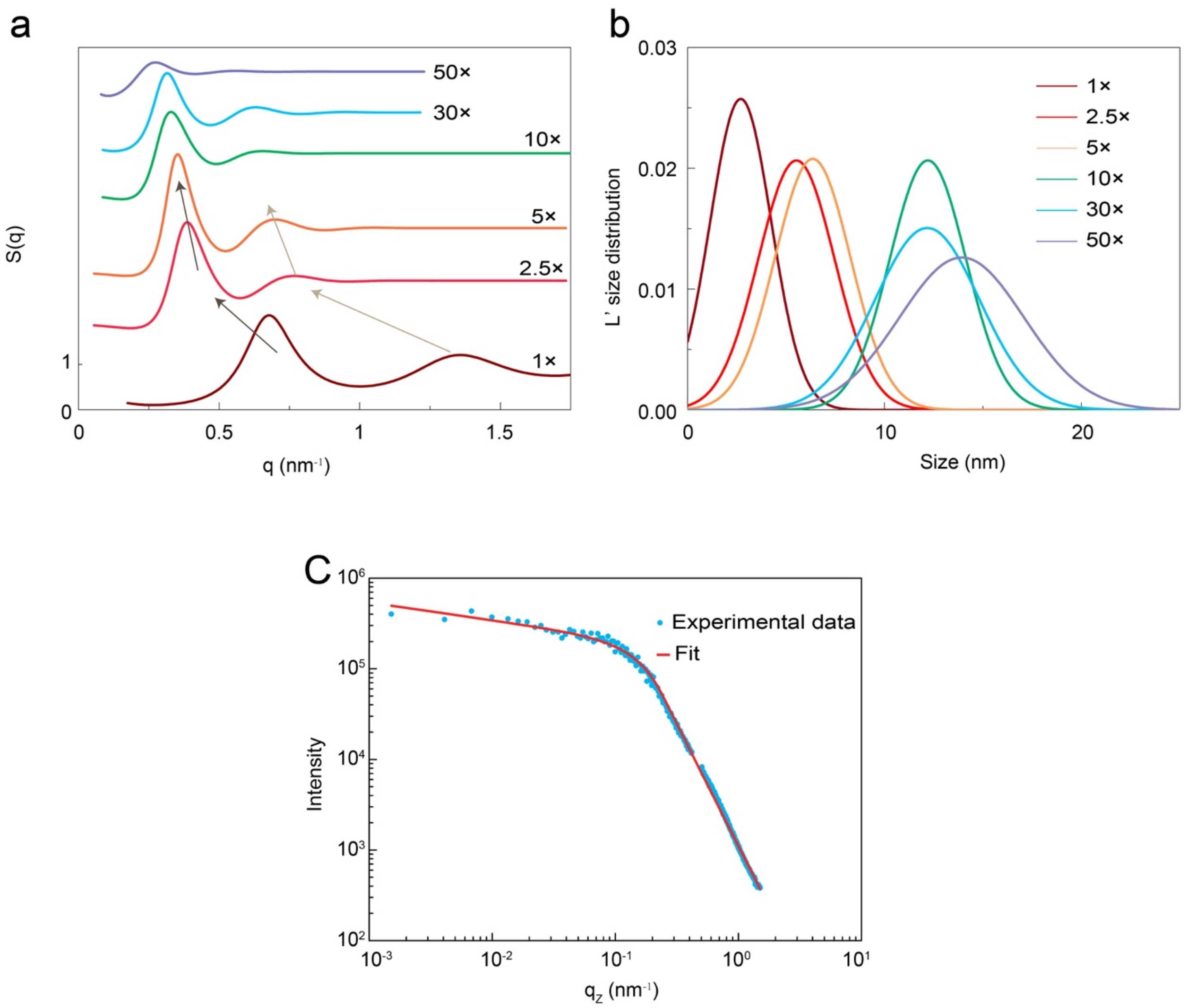

Supplementary Figure 11. The structure factor and size distribution analyzed by SAXS. a The structure factor $S(q)$ is modeled using the 1D para-crystal model, and describes how the units are stacked to form a lamellar superlattice. The curves are vertically shifted for clarity (except 1×). As draw ratio increases, the humps move to smaller **q**, suggesting larger length scales in the higher draw ratio films. The humps become less significant at higher draw ratios, suggesting more structural disordering as shown in Supplementary Figure 11b. **b** The length distribution of L' phases along the fiber direction at different draw ratios (L' is the length of crystal and transition region shown in Supplementary Figure 10). (**c**) Statistical analysis of fiber diameter, the fitting curve for 70× sample by Guinier-Porod model. The obtained diameter of nanofiber is ~11.1 nm.

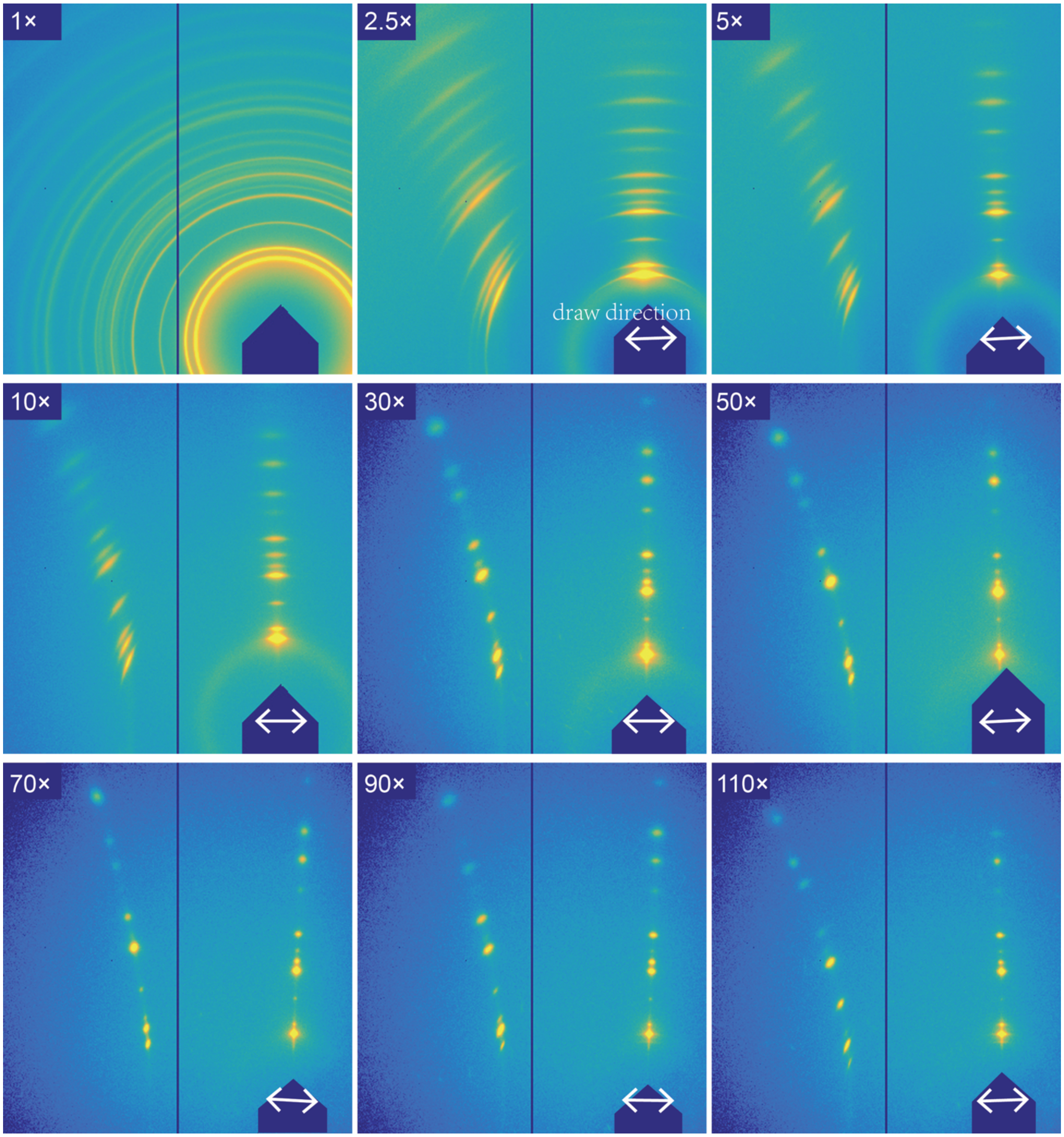

Supplementary Figure 12. WAXS patterns collected for 1× to 110× polyethylene films. The as-extruded (1×) film is isotropic, while the drawn films (from 2.5× to 110×) all show some anisotropic features. Detailed analysis can be found in Supplementary Note 4.

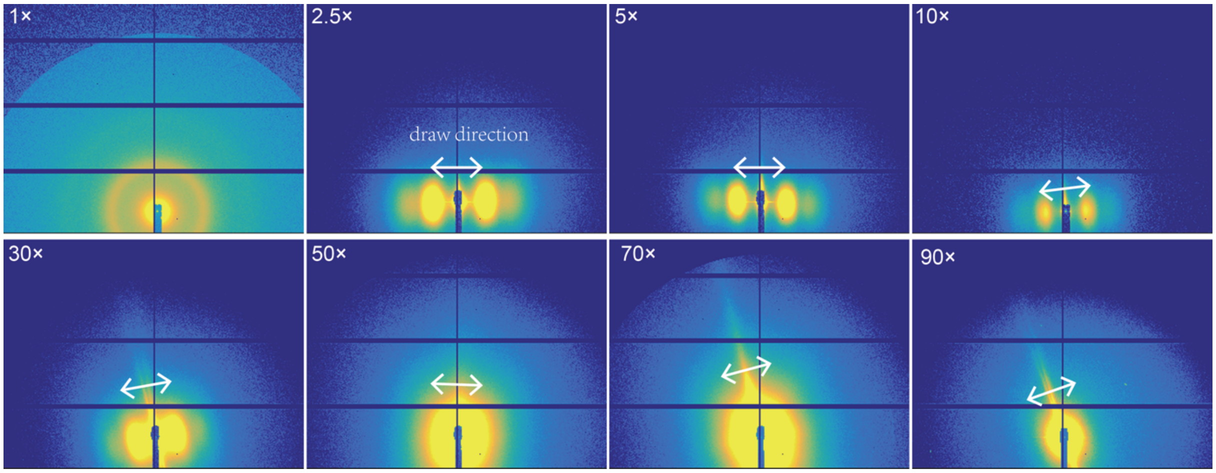

Supplementary Figure 13. SAXS patterns collected for 1× to 90× polyethylene films. The as-extruded (1×) film is isotropic, while the drawn films (from 2.5× to 90×) all show some anisotropic features. Detailed analysis can be found in Supplementary Note 4.

.

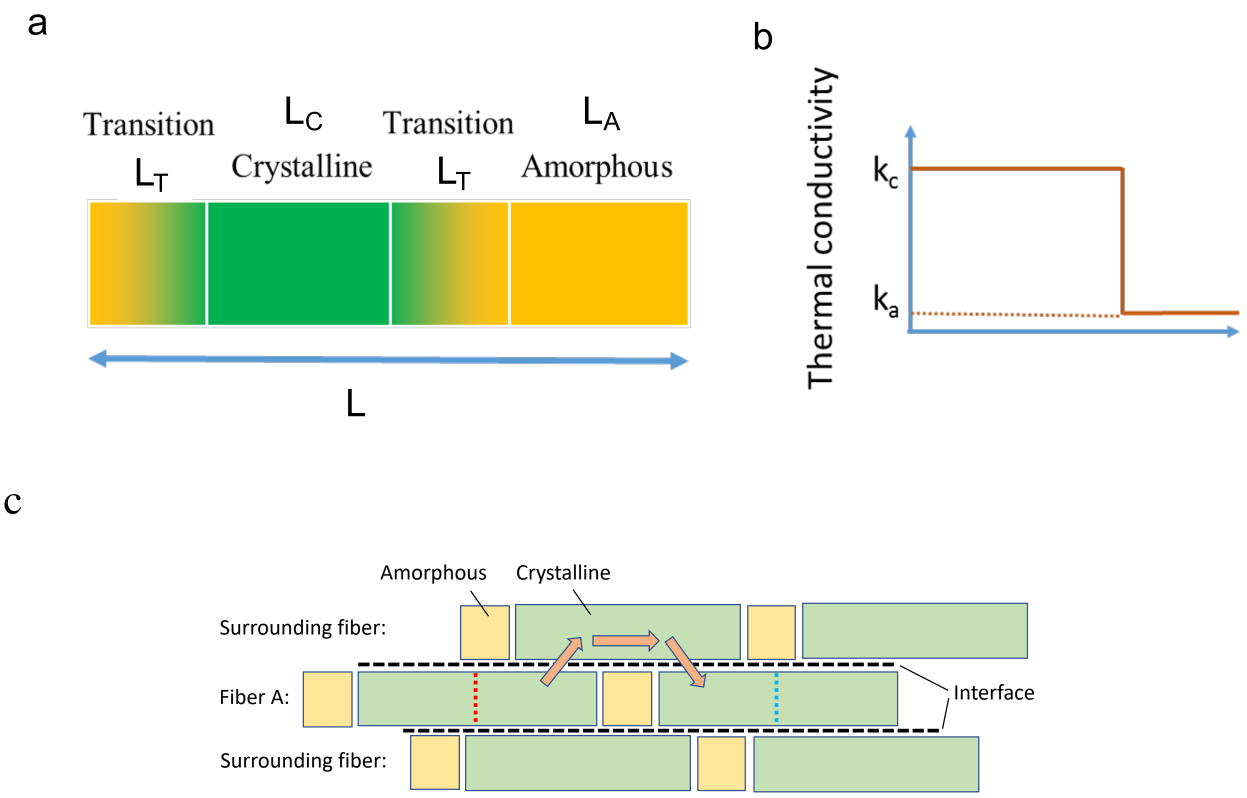

Supplementary Figure 14. Thermal conductivity model. a Schematic of the 1D model, a simplification of Supplementary Figure 10. **b** Modeled variation of thermal conductivity along chain direction in one unit cell, noting that the transition region has been included into the crystalline part by assuming that the former has a thermal conductivity equal to that of the latter. **c** Schematic for heat flow along the curved path (start at fiber A, flow into surrounding fibers, and then back to fiber A). While the path is drawn for one fiber in the surrounding, in reality multiple fibers can exist and has been taken into account in the analysis.

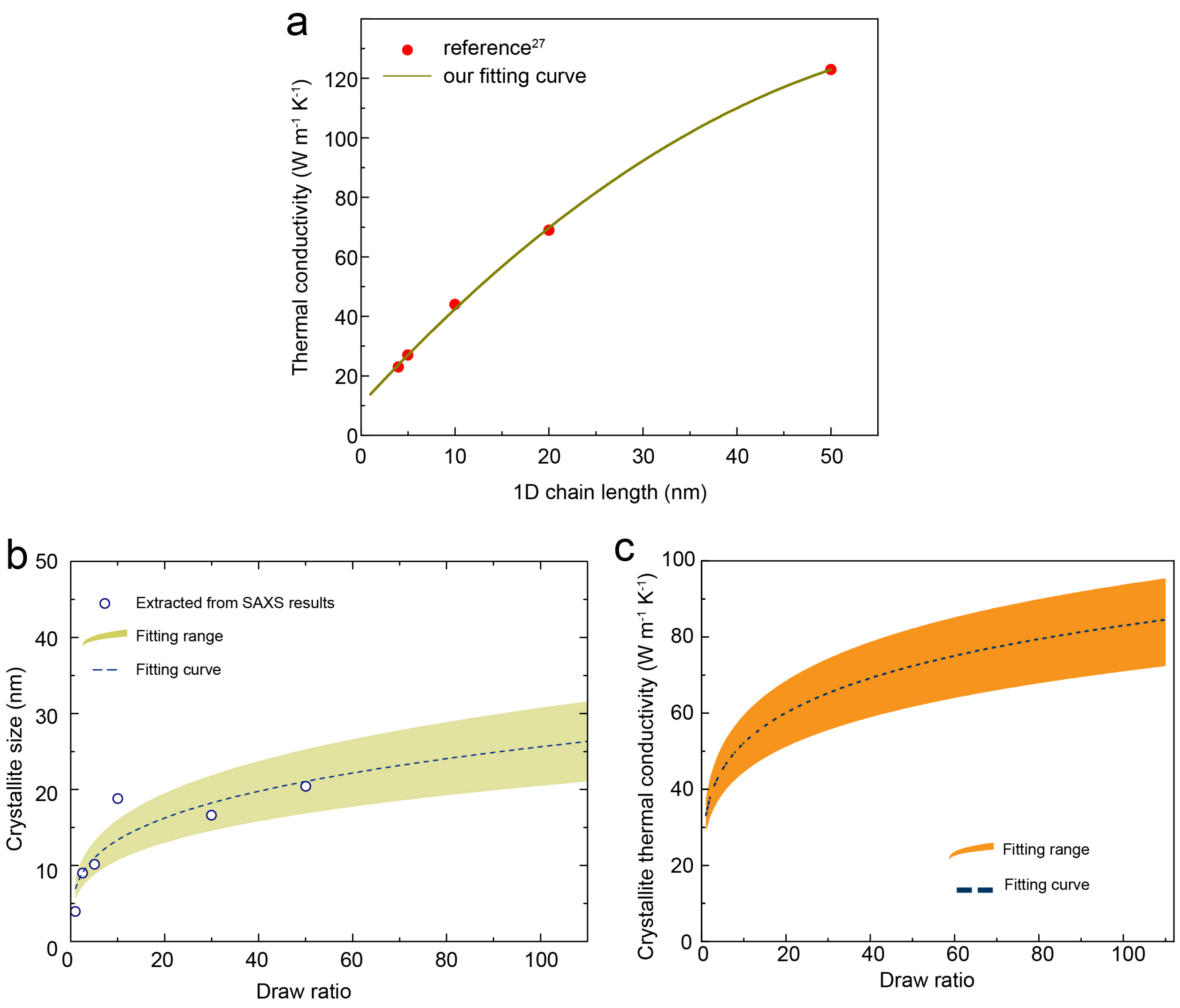

Supplementary Figure 15. Crystallite size and crystalline thermal conductivity model. a Thermal conductivity (along the chain direction) as a function of the 1D polyethylene chain length from literature[27]. Simulated data (dot) is from reference[27], and curve from fitting. **b** Crystallite size at different draw ratios. **c** Crystalline thermal conductivity at different draw ratios. The shaded regions are the fitted range, and the dashed line represents the fitting curve.

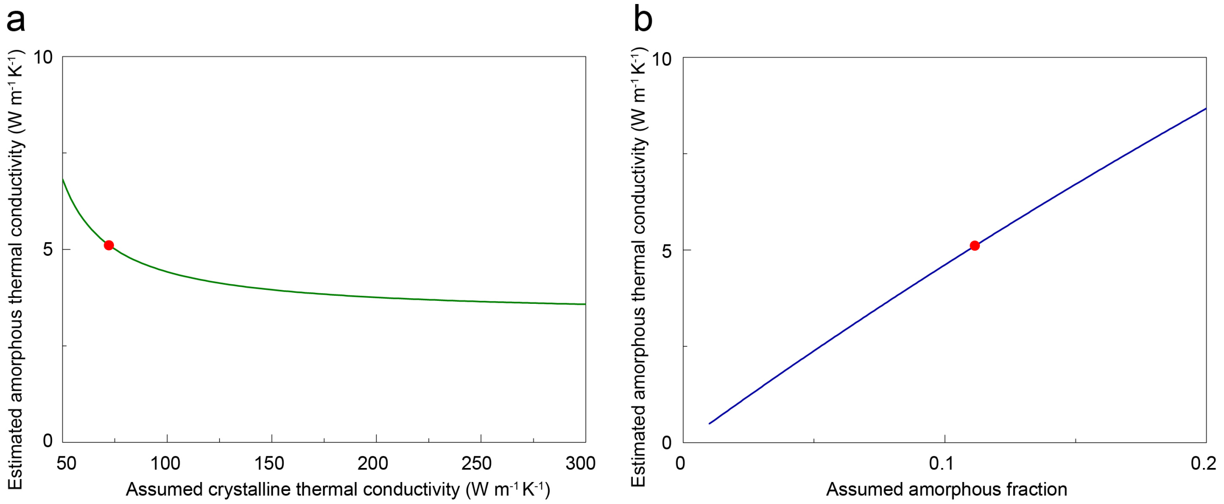

Supplementary Figure 16. Sensitivity analysis. Dependence of estimated amorphous phase thermal conductivity on (a) crystalline state thermal conductivity, and (b) amorphous fraction at 50× sample. The red point corresponds to the actual case while the green and blue line indicates the variation if those parameters are changed.

Supplementary References

1. Loomis, J. *et al.* Continuous fabrication platform for highly aligned polymer films. *Technology* **02**, 189–199 (2014).
2. Wang, X., Ho, V., Segalman, R. A. & Cahill, D. G. Thermal conductivity of high-modulus polymer fibers. *Macromolecules* **46**, 4937–4943 (2013).
3. Kraemer, D. & Chen, G. A simple differential steady-state method to measure the thermal conductivity of solid bulk materials with high accuracy. *Rev. Sci. Instrum.* **85**, 025108 (2014).
4. Kraemer, D. & Chen, G. High-accuracy direct ZT and intrinsic properties measurement of thermoelectric couple devices. *Rev. Sci. Instrum.* **85**, 045107 (2014).
5. Kraemer, D. *et al.* High thermoelectric conversion efficiency of MgAgSb-based material with hot-pressed contacts. *Energy Environ. Sci.* **8**, 1299–1308 (2015).
6. Sweet, J. N., Roth, E. P. & Moss, M. Thermal conductivity of Inconel 718 and 304 stainless steel. *Int. J. Thermophys.* **8**, 593–606 (1987).
7. Arpaci, V. S., Kao, S.-H. & Selamet, A. *Introduction to Heat Transfer*. (Prentice Hall).
8. Paddock, C. A. & Eesley, G. L. Transient thermoreflectance from thin metal films. *J. Appl. Phys.* **60**, 285–290 (1986).
9. Cahill, D. G. *et al.* Nanoscale thermal transport. *J. Appl. Phys.* **93**, 793–818 (2003).
10. Schmidt, A. J., Chen, X. & Chen, G. Pulse accumulation, radial heat conduction, and anisotropic thermal conductivity in pump-probe transient thermoreflectance. *Rev. Sci. Instrum.* **79**, 114902 (2008).
11. Cao, B., Sweeney, P. & Campbell, G. A. Infrared characteristics of thin polymer film: temperature measurement of polyethylene. *J. Plast. Film Sheeting* **6**, 153–161 (1990).

12. Xie, X. *et al.* Thermal conductivity, heat capacity, and elastic constants of water-soluble polymers and polymer blends. *Macromolecules* **49**, 972–978 (2016).

13. Bestiaansen, C. W. M. & Lemstra, P. J. Melting behaviour of gelspun/drawn polyolefins. *Makromol. Chem., Macromol. Symp.* **28**, 73–84 (1989).

14. Wunderlich, B. Specific heat of polyethylene single crystals. *J. Phys. Chem.* **69**, 2078–2081 (1965).

15. Litvinov, V. M. *et al.* Morphology, chain dynamics, and domain sizes in highly drawn gel-spun ultrahigh molecular weight polyethylene fibers at the final stages of drawing by SAXS, WAXS, and 1H solid-state NMR. *Macromolecules* **44**, 9254–9266 (2011).

16. Jiang, Z. GIXSGUI: a MATLAB toolbox for grazing-incidence X-ray scattering data visualization and reduction, and indexing of buried three-dimensional periodic nanostructured films. *J. Appl. Crystallogr.* **48**, 917–926 (2015).

17. Peacock, A. *Handbook of polyethylene: structures, properties, and applications*. (CRC Press, 2000).

18. Kasai, N. & Masao, K. *X-ray diffraction by macromolecules*. (Springer, 2005).

19. DeLongchamp, D. M., Kline, R. J., Fischer, D. A., Richter, L. J. & Toney, M. F. Molecular characterization of organic electronic films. *Adv. Mater.* **23**, 319–337 (2011).

20. Deutsch, M. Orientational order determination in liquid crystals by X-ray diffraction. *Phys. Rev. A* **44**, 8264–8270 (1991).

21. Feigin, L. A. & Svergun, D. I. *Structure analysis by small-angle X-ray and neutron scattering*. (Springer, 1987).

22. Hosemann, R. & Bagchi, S. N. *Direct analysis of diffraction by matter*. (North-Holland Publishing Company, Amsterdam, 1962).

23. Millane, R. P. & Eads, J. L. Diffraction by one-dimensional paracrystals and crystallography perturbed lattices. *Acta Cryst* **A56**, 497–506 (2000).

24. Lazzari, R., Leroy, F. & Renaud, G. Grazing-incidence small-angle x-ray scattering from dense packing of islands on surfaces: development of distorted wave Born approximation and correlation between particle sizes and spacing. *Phys. Rev. B* **76**, 125411 (2007).

25. Leroy, F., Lazzari, R. & Renaud, G. Effects of near-neighbor correlations on the diffuse scattering from a one-dimensional paracrystal. *Acta Crystallogr. A* **60**, 565–581 (2004).

26. Hammouda, B. A new Guinier–Porod model. *J. Appl. Crystallogr.* **43**, 716–719 (2010).

27. Wang, X., Kaviany, M. & Huang, B. Further improvement of lattice thermal conductivity from bulk crystalline to 1-D-chain polyethylene: a high-yet-finite thermal conductivity using first-principles calculation. *https://arxiv.org/abs/1701.02428 (2017)*.

28. Losego, M. D., Grady, M. E., Sottos, N. R., Cahill, D. G. & Braun, P. V. Effects of chemical bonding on heat transport across interfaces. *Nat. Mater.* **11**, 502–506 (2012).
